# Sustainable Cements with up to 80 wt% Calcined Common Clay: Challenges in Microstructure and Compressive Strength of Concretes

**DOI:** 10.3390/ma19112322

**Published:** 2026-05-31

**Authors:** Maximilian Panzer, Sebastian Scherb, Nancy Beuntner, Karl-Christian Thienel

**Affiliations:** Institute for Construction Materials, University of the Bundeswehr Munich, Werner-Heisenberg-Weg 39, 85579 Neubiberg, Germany; sebastian.scherb@unibw.de (S.S.); nancy.beuntner@unibw.de (N.B.); christian.thienel@unibw.de (K.-C.T.)

**Keywords:** calcined common clay, calorimetry, thermogravimetry, porosity, scanning electron microscopy images, mortar compressive strength, concrete compressive strength, carbonation, global warming potential

## Abstract

In this article, binders containing 0, 40 and 80 wt% calcined common clay are investigated in pastes, mortars and concretes. The investigation covers heat flow, microstructural aspects, compressive strengths, carbonation and sustainability calculations. The initially slow pozzolanic reactivity of the calcined common clay causes a decline of the 2-day strength exceeding its replacement level. As the pozzolanic reaction develops with time, concretes with 40 wt% binder replacement achieve up to 10% higher strengths than their reference at day 90. A further increase in the replacement to 80 wt% calcined clay yields concrete strengths that are at least 30% lower than the reference, because portlandite lacks for a complete pozzolanic reaction of the calcined common clay. This is counteracted by adding hydrated lime, which improves strength by up to 10 MPa. Microstructural investigations substantiate the strength findings. As the amount of hydrated phases declines and porosity rises, strength decreases, and vice versa. Replacing 40 or 80 wt% of Portland cement by calcined clay reduces the Global Warming Potential of concretes by nearly a quarter or half. The concretes with 40 wt% replacement exhibit the best strength eco-efficiency from day 28 onwards, while the concretes with 80 wt% replacements achieve a strength eco-efficiency comparable to that of the reference concretes.

## 1. Introduction

Calcined clay is used in many publications in combination with limestone powder in a ratio of 2:1 as supplementary cementitious material. The resulting binder is often referred to as LC^3^ which has a clinker proportion of 50 wt% [1,2,3]. With the usually used calcined kaolinitic clays [1], a higher proportion than 30 wt% is not advisable due to their high consumption of portlandite in their strong pozzolanic reaction [4,5,6,7]. A further increase would require more portlandite than is available, since it is provided by the silicate clinker reaction of an in parallel declining clinker in the binder. Both effects will result in an incomplete reaction of the calcined kaolinitic clay. Even with a binder consisting of 80 wt% Portland cement and 20 wt% metakaolin, there is hardly any portlandite left at 28 days [8]. Its content is approximately as high as observed with a 40 wt% replacement by metaillite or metasmectite [8]. But the reactivity of these clay minerals is not as high as that of metakaolinite [7,9,10,11,12]. Common clays [13,14], which contain all three clay minerals and inert components, offer a compromise between portlandite consumption and reactivity [5,15,16,17,18].

An important reason for using limestone in LC^3^ is its synergistic effect with calcined clay. The formation of carboaluminates and the preservation of ettringite reduce porosity and thus increase strength [15,19,20]. However, limestone is as an inert substitute which acts physically [2,21] and not pozzolanic as calcined clays. In addition to the C-S-H formation of classic pozzolans, calcined clays produce aluminum-rich reaction products such as carboaluminates, C-A-S-H phases, or strätlingite [7,15,22,23]. More hydrate phases are formed with kaolinitic clays than with 2:1-dominated (illitic, smectitic) clays [8,24,25]. Usually, the gypsum content in LC^3^ is set to 5 wt% to satisfy the sulfate carrier requirement of kaolinitic clays, which is higher than for the 2:1-dominated clays [10,19,26]. As can be seen in heat flow measurements of blended cements [19,27,28,29], calcined clay causes the aluminate peak to occur earlier due to its filler effect [30] and adsorption of sulfate carrier [19]. The addition of a sulfate carrier counteracts this and normalizes the early hydration of the blended cement [10,19,26,31,32]. Without sulfate adjustment, the early strength of the blended cement is decreased which has a significantly greater impact with kaolinitic clays than with 2:1-dominated clays [22,25,26,32].

Mortars and concretes with LC^3^ reach at least the same 28-day compressive strengths than the reference made with Portland cement [22,29,33,34,35]. But this is not achieved at 2 days, as metakaolinite needs time for its pozzolanic reaction, even though it reacts faster than the other calcined clay minerals [5,6,7]. A further clinker reduction below 50 wt% using calcined kaolinitc clay decreases the strength, but is only demonstrated in mortars [29,36,37] and not in concretes. Reducing the clinker factor from 0.5 to 0.2 in favor of calcined kaolinitic clay and limestone powder leads to a 50–70% strength reduction at 28 days, resulting in a compressive strength of only about 20 MPa [29,37]. To counteract the high portlandite consumption of calcined kaolinitic clays, extra hydrated lime (Ca(OH)_2_) can be added to ensure a complete pozzolanic reaction of the metakaolinite [4,29]. This approach improves their low mortar compressive strengths at 28 days by 5 to 15 MPa [29,36].

Beyond strengths, ecological considerations are becoming significant. The very high Global Warming Potential (GWP) of hydrated lime must be considered [13] since more CO_2_ is emitted during its production than the material produced. This is not even the case with Portland cement, which has a 30% lower GWP [38]. Replacing Portland cement by calcined clay reduces the GWP many times and the GWP of limestone powder is neglectable [38]. In sum, the GWP from approx. 900 kg CO_2_ per ton Portland cement is reduced to less than 600 kg CO_2_ per ton LC^3^ [37,39]. Low-carbon concretes are defined based on their GWP for a given compressive strength [40,41]. Since both parameters are favorable for concretes with calcined clay as a binder component, they outperform classic concretes with Portland cement and are at a similar level to current concretes produced with a low clinker cement (CEM III/A and CEM II/C-M) [29,42]. The optimal balance between GWP and strength is governed by the replacement level of calcined clay. Beyond a certain level, concrete strength decreases to an extent that can no longer be offset by the reduced GWP.

A consequence of using calcined clays as part of the binder is the faster carbonation of mortars and concretes, which is more pronounced the higher their replacement level and kaolinite content [42,43,44,45]. The pozzolanic reaction of the calcined clays consumes portlandite and thus jeopardizes corrosion protection. The carbonation of a sample is also influenced by its porosity, as CO_2_ diffusion depends on it. While low replacements (up to 30 wt%) of calcined clay hardly change the porosity [15,46,47], it increases with very high replacements [29].

Previous research has mainly targeted kaolinitic clays, overlooking common clays. The latter are far more prevalent in Europe and other parts of the world [48,49] and deserve more attention. While the use of kaolinitic clays restricts the reduction in the clinker proportion in the binder, the current minimum of 50 wt% clinker does not necessarily set the lower limit for a calcined clay blended cement. For this reason, this article investigates binder substituted by up to 80 wt% calcined common clay and addresses the following research questions for the first time: what strengths do concretes achieve with a very high proportion of calcined common clay in the binder and how are they assessed in terms of sustainability? How does the microstructure change with a very high replacement by calcined common clay? Therefore, 20 different concretes were produced and tested for compressive strength at 2 to 90 days, differing in terms of water-to-binder (w/b) ratio (0.40–0.50), binder content (240–450 kg/m^3^) and binder composition. Portland cement was replaced by 40 and 80 wt% calcined common clay, which was also partly combined with up to 40 wt% limestone powder and up to 20 wt% hydrated lime. In addition, the compressive strengths in mortar were also determined for the various binder compositions at 2 to 90 days. The sustainability assessment of the concrete strengths was based on the GWP of the concretes. The carbonation of selected concretes was examined up to 168 days and images of their microstructure were taken with a scanning electron microscope. For the different binder compositions, the heat flow was determined calorimetrically up to 7 days, the amount of hydrate phases was analyzed thermogravimetrically at 2 to 56 days, and the porosity was measured at 28 days for hardened pastes (w/b = 0.50).

## 2. Materials and Methods

### 2.1. Materials and Characterization

#### 2.1.1. Materials

Table 1 presents the mineralogical composition provided by the producer, chemical composition and physical parameters of the Portland cement CEM I 42.5 N used. The parameter d’ refers to the particle diameter corresponding to 63.2 vol% passing in accordance with DIN 66145 [50]. A complete illustration of the particle size distribution is given in Supplementary Figure S1. The methods used for material characterization are described in Section 2.1.2.

Table 2 summarizes the mineralogical and chemical composition, physical parameters and reactivity of the common clay (CT7), limestone powder (LSP) and hydrated lime (CH). The 66 wt% clay minerals in the raw clay are different phyllosilicates, allowing the clay to be classified as common clay. Components described as “Others” are dolomite, feldspar, rutile and pyrite. The clay was calcined in an industrial rotary kiln at 750 °C and ground in an industrial roller mill [51]. In this form, the clay is called CT7 and is used for investigations in this study exclusively. Limestone powder was taken as delivered. A commercially available Cl 90 was used for the hydrated lime addition. The particle diameters at 10, 50 and 90 vol% passing are denoted as d_10_, d_50_ and d_90_. The particle size distributions of the calcined clay, limestone powder and hydrated lime are added in Supplementary Figure S1. The reactivity of the calcined common clay is given in the form of cumulative heat release at 24 and 168 h in the R^3^ test and the solubility of Al and Si ions after 20 and 168 h of elution in 10% NaOH solution.

Dry aggregates from a local supplier were used in all concretes with a grading curve A/B16 according to DIN 1045-2 [52] and a raw density of 2.74 g/cm^3^ measured according to DIN EN 1097-6 [53]. A PCE superplasticizer was chosen to adjust workability, which has a solid proportion of 39.5 wt%.

The relative binder compositions for the blended cement pastes, mortars and concretes are shown in Table 3. The nomenclature of the binder specifies the relative composition in wt%: cement/calcined clay + limestone powder/hydrated lime. No number is written if limestone powder or hydrated lime is not contained.

Although the hydrated lime is termed an “addition”, it is always considered as part of the 100 wt% binder. Therefore, the added amount of hydrated lime is subtracted proportionally from the reference cement and the replacing calcined clay. Even though the calcined clay proportion is reduced due to the hydrated lime addition, the replacement level continues to be stated as 40 or 80 wt%.

Pastes, mortars and concretes were each produced with the various binder compositions from Table 3. Table 4 shows their water-to-binder (w/b) ratio and binder content (b). Heat flow measurements, thermogravimetric analysis and porosity examinations were carried out with the pastes. Mortar prisms were tested for their compressive strength. Concretes were produced with all binder compositions at a binder content of 360 kg/m^3^ (w/b = 0.50) and without limestone powder or hydrated lime at a binder content of 300, 240 (each w/b = 0.50) and 450 kg/m^3^ (w/b = 0.40). On the one hand, the binder and water contents are reduced to maintain a constant w/b ratio (0.50), and on the other hand, the binder content is increased while the water content remains constant (w/b ratio falls to 0.40). All concretes were tested for their compressive strength, some selected concretes with 360 and 240 kg binder/m^3^ additionally for their carbonation and portlandite contents, and in the scanning electron microscope.

The concrete compositions calculated with an assumed air content of 1.5 vol% are listed in Table A1 and Table A2. The w/b ratio of the concretes (0.40, 0.50) does not consider the amount of superplasticizer added. Although the water content of the superplasticizer is accounted for, the w/b ratio hardly changes due to the low superplasticizer dosages and is therefore not considered in the following. The effective w/b ratio (called w/b_eff_) is also listed, which reflects the water content of the superplasticizer and hydrated lime (Ca(OH)_2_), and only the reactive components in the binder such as cement, calcined clay minerals and quicklime (CaO).

#### 2.1.2. Methods for Material Characterization

The methods used to characterize the materials (cement, calcined clay, limestone powder, hydrated lime) are presented as follows. The particle density was measured using a Helium pycnomatic ATC (POROTEC GmbH, Hofheim, Germany) in accordance with DIN EN ISO 17892-3 [54] and the chemical composition was tested by inductively coupled plasma optical emission spectrometry (ICP-OES) in accordance with DIN EN ISO 11885 [55]. The BET specific surface area (BET SSA) was determined with SA-9601 MP (Horiba Instruments Incorporated, Irvine, CA, USA) according to DIN ISO 9277 [56] and the particle size distribution via static laser light diffraction using a Bettersizer S3 Plus (3P Instruments GmbH, Odelzhausen, Germany). The water demand of cement to reach standard stiffness was obtained in line with DIN EN 196-3 [57] and its Blaine specific surface area (Blaine SSA) in line with DIN EN 196-6 [58]. The compressive strength of the cement at 2 days (f_2d_) and 28 days (f_28d_) was determined on mortar prisms in accordance with DIN EN 196-1 [59].

The Puntke method [60] was applied for the water demand of the calcined clay, limestone powder and hydrated lime. The reactivity of the calcined clay and limestone powder was quantified as cumulative heat release in the R^3^ test according to DIN EN 17979 (test method A) [61] with a TAM Air isothermal calorimeter (TA Instruments). The solubility of aluminum (Al) and silicon (Si) ions of the calcined clay and limestone powder was measured after 20 h of elution in 10% NaOH solution, additionally after 168 h for the calcined clay [62]. X-ray diffraction (XRD) analyzed the mineralogical composition of the raw clay, limestone powder and hydrated lime on powder samples prepared by side-loading. The clay minerals were identified on oriented mounts of the < 2 μm fraction prepared by the glass slide method [63] in air dried and glycolated condition. The detailed procedure is described in [5]. XRD was made in a PANalytical Empyrean diffractometer (Malvern Panalytical GmbH, Kassel, Germany) equipped with a BraggBrentano^HD^ monochromator and a PIXcel^1D^ detector at 40 kV and 40 mA using CuKα radiation (1.5406 Å). The data evaluation was conducted with Profex-BGMN (version 5.2.8) [64,65] using Rietveld refinement [66,67].

### 2.2. Methods Used in Investigations

#### 2.2.1. Heat Flow Measurements

The early hydration kinetic of the different binder compositions was analyzed calorimetrically. First, 5 g binder was mixed by hand for 30 s. Then, 2.5 g water was added and the paste was stirred manually and externally for one minute. Approx. 3 g of paste was placed into sample crucibles and inserted into the TAM Air isothermal calorimeter (TA instruments, New Castle, DE, USA) at 23 °C. The reference samples in the calorimeter are a mixture of 2 g quartz sand and 1 g water. The heat flow was monitored for 168 h and normalized to 1 g of binder. Every binder composition was measured twice and the results were averaged. The hydration heat was calculated by integrating the heat flow over time from 0.5 to 168 h. The initial value was chosen because the early heat flow was not reproducible due to external mixing. By that time, all samples had completed the initial period and entered the rest period.

#### 2.2.2. Thermogravimetric Measurement

Thermogravimetric analyses were performed on the hardened blended cement pastes to quantify their hydrate phases at 2, 28 and 56 days. The same blended cement pastes were produced identically as for the heat flow measurements. Following 24 h of airtight dry storage at 23 °C, the blended cements were kept in distilled water in crushed form at 23 °C until the desired hydration age was reached. Then, the samples were ground by hand in a mortar and their hydration was stopped with diethyl ether and isopropanol. After 10 min in diethyl ether and 30 min in isopropanol, they were dried at 60 °C for 60 min. Equal amounts of sample, again ground by hand, were analyzed in the STA 449 F5 Jupiter (Netsch-Gerätebau GmbH, Selb, Germany) at a heating rate of 10 °C/min in the range from 25 to 900 °C.

The dehydration temperatures of the hydrate phases used for this investigation are reported in [15]. Dehydration up to 400 °C is split into three stages: 25–140 °C (stage 1), 140–190 °C (stage 2), 190–400 °C (stage 3) [15]. Ettringite and C-S-H phase dehydrate in stage 1 and monophases (AFm) such as hemi- and monocarboaluminate, monosulfoaluminate and strätlingite in stages 2 and 3. The mass loss of the hardened paste in the individual stages (temperature ranges: T_1_–T_2_) is called bound water H_2_O_T1–T2_ [wt%]. Portlandite (Ca(OH)_2_) dehydrates at about 450 °C. Its temperature ranges (T_a_–T_b_) are determined for every individual hardened paste with the tangent method of Marsh and Day [68]. The portlandite content is calculated on the basis of its dehydration H_2_O_Ta–Tb_ in accordance with Equations (1) and (2) [69]. The latter is related to cement amount by the proportion z of cement in the binder. The ratio of the molar mass of portlandite M_Ca(OH)2_ to that of water M_H2O_ is 4.113, and the portlandite content is referred to the sample mass at 400 °C.

(1) $${Ca\left(OH\right)}_{2}\left[wt\%\right]=\frac{M_{{Ca(OH)}_{2}}}{M_{H_{2}O}}\cdot \frac{H_{2}O_{T_{a}–T_{b}}}{1-\frac{{H_{2}O}_{25–400\;{}^{\circ}C}}{100\;wt\%}}=4.113\cdot \frac{H_{2}O_{T_{a}–T_{b}}}{1-\frac{{H_{2}O}_{25–400\;{}^{\circ}C}}{100\;wt\%}}$$

(2) $${Ca(OH)}_{2}[\frac{g}{100\;g\;cement}]=\frac{{Ca(OH)}_{2}\;[wt\%]}{z}$$

Inner fragments of concretes were measured thermogravimetrically at 168 days (1 day in mold, 27 days in water, 140 days dry at 23 °C) without hydration stop (10 °C/min heating rate up to 900 °C) to investigate their portlandite content. Care was taken during sample preparation to remove as many aggregates as possible.

Each sample of hardened paste and concrete was measured twice, and the results were averaged.

#### 2.2.3. Porosity Measurement

The porosity of the hardened pastes at 28 days was determined by mercury pressure porosimetry in accordance with DIN 66133 [70]. The same blended pastes were produced and stored in the same manner as those used for the thermogravimetric analysis. After storage in isopropanol for several days and subsequent drying at 60 °C, cubic pieces were analyzed in the Thermo Scientific Pascal Series mercury porosimeter (ThermoFisher Scientific, Germering, Germany) in the low-pressure unit (400 kPa) for pore radii down to 2 µm and in the high-pressure unit (400 MPa) for pore radii down to 2 nm. The results from two measurements in each case were averaged. The porosity of the hardened pastes is classified into four pore size ranges: >50 µm (air voids), 50–1 µm (capillary pores), 1–0.03 µm (microcapillary pores) and <0.03 µm (gel pores) [71].

#### 2.2.4. Compressive Strength Tests

Mortar prisms were produced in accordance with DIN EN 196-1 [59] and tested at 2, 7, 28, 56 and 90 days to determine the compressive strength of the different binder compositions. The mortar contains 1350 g standard sand, 225 g water and 450 g blended cement, corresponding to a w/b ratio of 0.50. Prior to automatic mixing with sand and water at a defined time and speed, the blends were manually homogenized for one minute. Superplasticizer was not added to the mortar. After molding with constant compaction, the mortar prisms were stored in the mold for 24 h (20 °C, 95% relative air humidity) and subsequently in water (20 °C) until testing using the universal testing machine TTM 200 (Form + Test Seidner + Co. GmbH, Riedlingen, Germany). The Activity Index of the mortar (AI_M_) was calculated by relating the compressive strengths (f_M_) of the blended cement to that of the reference cement at the same age. Except for four compressive strength values used at 28 days, two values were averaged for each test age.

The compressive strength of concretes was tested using 15-cm cubes, which were stored in water (20 °C) until the test after one day in the mold (20 °C). The production, storage and testing were carried out according to DIN EN 12390-2 [72] and DIN EN 12390-3 [73]. First, the aggregates were mixed in a compulsory mixer for 30 s. Then, the binder was added and they were mixed together for 30 s. During mixing, water and superplasticizer were added and the concrete was mixed for two minutes. The amount of superplasticizer was chosen to achieve a soft consistency (F3) [74] in the slump test (DIN EN 12350-5 [75]) immediately after mixing. Finally, the concrete was filled into the molds in two distinct layers, while compaction was carried out on a vibrating table with a constant frequency of 4500 rpm for 30 s for each layer. Three concrete cubes were tested for the compressive strength (f_C_) and averaged for each test age (2, 28, 56, 90 days) using the universal testing machine Alpha 3000 (Form + Test Seidner + Co. GmbH, Riedlingen, Germany). The Activity Index of the concrete (AI_C_) relates the concrete compressive strengths with blended cements to the compressive strengths of the reference concrete (100/0) for each individual binder content at the same age.

#### 2.2.5. Scanning Electron Microscopy Images

Scanning electron microscopy (SEM) images of hardened concretes (360 kg binder/m^3^) with different binder compositions were made at 90 days to study the interfacial transition zone and hydrate phases. The concretes, which were filled into prism molds for carbonation tests, were hydrated for 1 day in the mold, 27 days in water and 62 days dry at 23 °C. Immediately after breaking the concrete prisms, flat pieces were gold coated and analyzed in a Zeiss EVO LS 15 scanning electron microscope (Carl Zeiss Microscopy Deutschland GmbH, Oberkochen, Germany) with LaB6 cathode and SE detector under high vacuum at a voltage of 20 kV. The magnification varies from 2000 to 20,000.

#### 2.2.6. Carbonation Test

The carbonation resistance of different binder compositions was carried out on concretes with a binder content of 240 and 360 kg/m^3^ until 168 days. DIN EN 12390-10 [76] was used as a guideline for the tests. The concretes produced for the compressive strength tests were filled into molds measuring 40 mm × 40 mm × 160 mm and compacted. After 1 day in the mold and 27 days in water (23 °C), the concrete prisms were stored dry in the laboratory under natural conditions (23 °C and 40% humidity). In an accelerated test, the carbonation of the concretes with 80 wt% calcined clay would occur so rapidly that it could not be measured over time. At the time of dry storage, the carbonation measurement began (t = 0) and discs of the prisms were broken off via the tensile splitting test for the first time and the fresh surface was sprayed with an indicator solution. This was repeated initially every 14 and later every 28 days. At each test age, the depth of the carbonation d_k_ was measured on three prisms at three points on each side and the twelve values were averaged. The carbonation speed k_c_ is calculated from the carbonation depth d_k_ at the last test age t or at the time of complete carbonation, if this is achieved before the end of the test:

(3) $$k_{c}=\frac{d_{k}\;[mm]}{\sqrt{t}\;[\sqrt{a}]}$$

#### 2.2.7. Calculations for Assessing Sustainability

In addition to compressive strength and carbonation behavior, the concretes are also analyzed for sustainability. For this purpose, the Global Warming Potential (GWP_C_) of the concrete is calculated using Equation (4). The mass of CO_2_ required to produce 1 m^3^ of concrete is calculated by multiplying the mass of the individual components (m_i_) by the mass-related CO_2_ emissions for their production (GWP_i_). The latter is given in Table 5 for the materials used [13,38,77]. The CO_2_ emissions from the raw material and fuel used in production are considered, but not those from transportation. The Integrated Strength Eco-Efficiency (I-SEE) coefficient [78] weighs the cube compressive strength of the concrete f_C_ sustainably with reference to the GWP_C_ (Equation (5)).

(4) $${GWP}_{C}=\sum_{i=1}^{n}m_{i}\cdot {GWP}_{i}$$

(5) $${I-SEE}_{C}=\frac{f_{C}}{{GWP}_{C}}$$

In order to finally classify low-carbon concretes, an adequate margin of 9 MPa (DIN EN 206-1 [79]) and concrete strength classes (DIN EN 1992-1-1 [80]) are considered for the conversion of the average cube strengths from the test into the characteristic cylinder strengths.

## 3. Results

The results of the various tests are presented below and will be discussed together in Section 4, as a full interpretation of a finding requires investigations conducted at a later stage.

### 3.1. Investigations on Pastes

#### 3.1.1. Heat Flow Measurements

**Figure 1:**
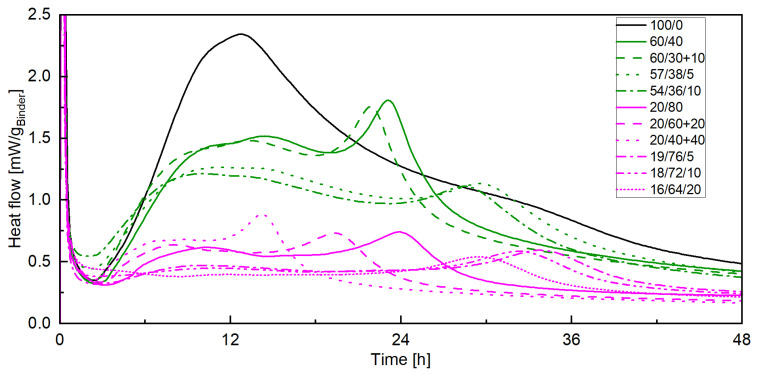
Heat flow of the pastes with different binder compositions.

The heat flow of the pastes up to 48 h is illustrated in Figure 1 and their hydration heat at 48 and 168 h is given in Table 6. The reference cement yields permanently the highest values, standing out with the silicate peak at 12 h and aluminate peak at 33 h. Accordingly, its hydration heat is also highest. Even after 48 h, no blended cement exhibits such an increase in hydration heat as the reference cement. A replacement by 40 wt% CT7 accelerates the aluminate peak but occurs at the same time as with 80 wt% CT7 (24 h). The decrease in heat flow with increasing replacement level is reflected in the hydration heat, since 60/40 has 20% and 20/80 has 60% lower values as the reference cement. The use of limestone powder accelerates the aluminate peak of its blended cement and further reduces the hydration heat, as also reported in [81]. The addition of hydrated lime delays the aluminate peak of the calcined clay blends and significantly reduces the hydration heat. Exceptions to the latter are the 5 and 10 wt% additions at the “80 wt%” replacement level, which have similar hydration heats as the reference cement. The results for hydrated lime addition are in line with [29].

The blends with “80 wt%” do not even have half the hydration heat of the reference cement at 7 days. When tested in accordance with the standard [82,83], it can therefore be expected that the requirement for the hydration heat in DIN EN 14216 [84] will be met to be classified as a very low heat special cement.

#### 3.1.2. Thermogravimetric Analysis

**Figure 2:**
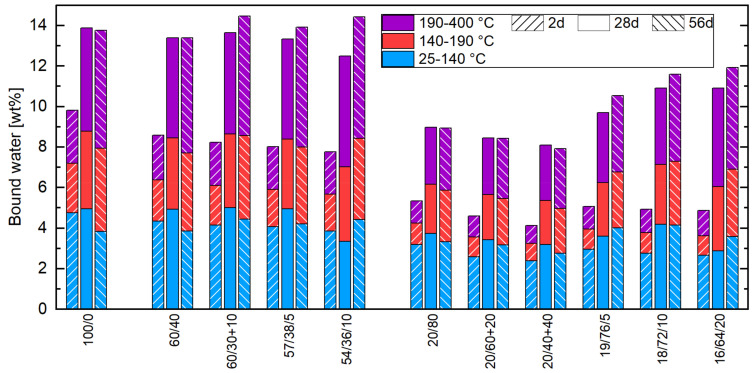
Bound water content of the hardened pastes with different binder compositions at 2, 28 and 56 days.

The bound water content of the various hardened pastes at 2, 28 and 56 days is summarized in Figure 2 (Supplementary Table S1). Most hydrate phases form within the first 28 days and hardly any after that. No greater quantity differences occur at 28 and 56 days between the reference cement and the blends with “40 wt%” replacement, but the blends have fewer hydrate phases at 2 days. Their individual distribution of the bound water amount is similar to that of the reference cement at every age. It is dominated by the dehydration up to 140 °C at 2 days, in which the amount hardly changes until day 56. This impression can be misleading, because the ettringite (AFt) formed reacts further to monosulfoaluminate (AFm) [15,33], which dehydrates above 140 °C. The increasing C-S-H phases formation during this time compensates for this. After 2 days, the amount of AFm phases (dehydration between 140 and 400 °C) increases significantly. The results of the 40 wt% CT7 blended cement are understandable, as the literature shows at similar replacement levels, that blends with 1:1-dominated clays have more bound water at 28 days than the reference cements, while blends with 2:1-dominated clays have less [8,24,25].

The bound water content in the blends with “80 wt%” replacement is significantly lower than that of the other binder compositions. At 2 days, approx. 50% is reached and later approx. 60%. The latter is higher for binders with hydrated lime addition because more AFm phases are present. The use of limestone powder reduces the bound water content as expected. With limestone powder, fewer hydrate phases are formed.

**Figure 3:**
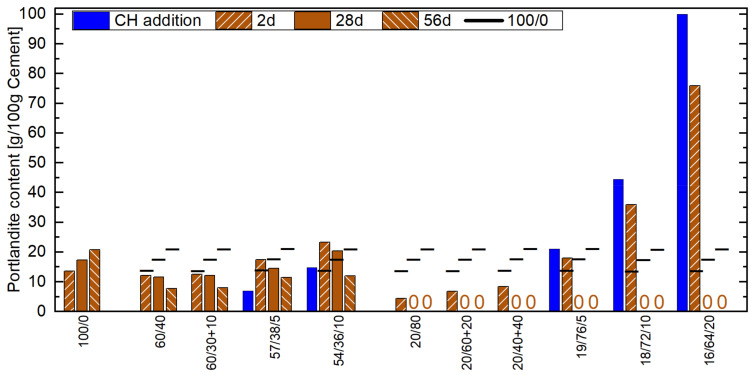
Portlandite content of the hardened pastes with different binder compositions at 2, 28 and 56 days. The short black lines indicate the portlandite content of 100/0 at the respective age, whereas the long blue column represents the portlandite content from hydrated lime addition alone.

The portlandite content of the blends (Figure 3) is normalized to the Portland cement content according to Equation (2). The reference to the entire binder (Equation (1)) reduces the values by the individual Portland cement proportion (Supplementary Table S1). While the portlandite content of the reference cement (100/0) increases with age during clinker hydration, it decreases in the blends with “40 wt%” replacement. Portlandite is no longer present in blends with “80 wt%” replacement after 2 days. A linear extrapolation from 100/0 and 60/40 to 20/80 is possible when referring to the binder (Equation (1)), because the portlandite content falls proportionally from 13.6 and 7.3 to 0.9 wt% at 2 days. Accordingly, a portlandite deficiency of 20/80 was expected at 28 days, since the portlandite content drops from 17.4 (100/0) to 7.0 wt% (60/40). Portlandite would only be present up to a replacement by 65 wt% CT7 based on the aforementioned results. In comparison, just 30 wt% metakaolin is sufficient in a blend for the complete consumption of portlandite at 28 days, but this can be counteracted by the addition of hydrated lime [4]. In this study, only the blends with hydrated lime addition do not have a lower portlandite content than the reference cement at 2 days. However, their portlandite content is always below the theoretical values. The hydrated lime addition at 16/64/20 alone would lead to a portlandite content of 100 g/100 g cement (blue column in Figure 3). Portlandite cannot be detected in the blends with “80 wt%” replacement at a later age, even with this high addition.

#### 3.1.3. Porosity Examinations

**Figure 4:**
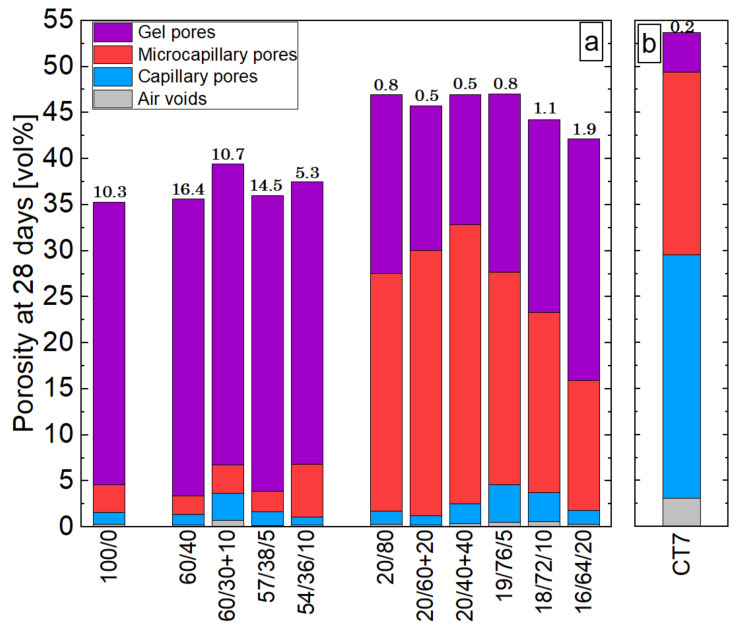
Porosity and its distribution of the hardened pastes at 28 days (**a**) and CT7 as solid (**b**). It is divided into air voids (>50 µm), capillary pores (50–1 µm), microcapillary pores (1–0.03 µm) and gel pores (<0.03 µm) [71]. The value above the column is the volume ratio of gel to microcapillary pores.

**Figure A1:**
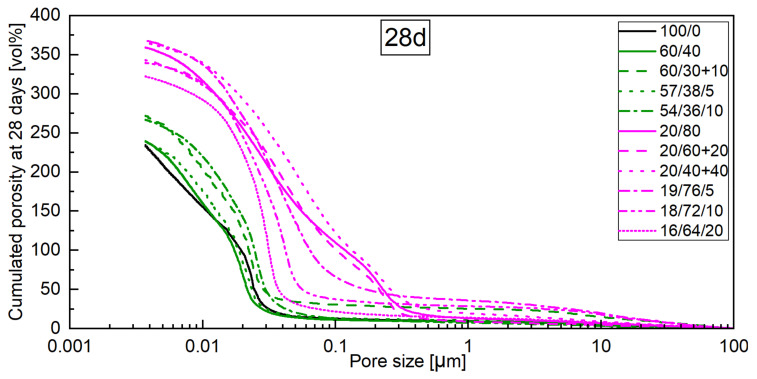
Cumulated porosity of the hardened pastes as a function of the pore size at 28 days.

The porosity and its distribution in the hardened pastes at 28 days are given in Figure 4a and Figure A1 (Supplementary Table S2). The total porosity of the reference cement remains almost unchanged for “40 wt%” replacement and in both cases the volumes of gel pores are many times higher than that of microcapillary pores. The situation is drastically different with “80 wt%” replacement, as the total porosity rises by approx. 10 vol%. Additionally, the volume of microcapillary pores increases, while that of gel pores decreases, leading to a low ratio of gel to microcapillary pores. The use of limestone powder further accentuates this trend, but the addition of hydrated lime counteracts it, so that the ratio increases again. Overall, their total porosity is reduced by hydrated lime addition, as in [29]. The porosity of the CT7 as a solid was also determined and shows a significantly higher total porosity and coarser pore structure (Figure 4b).

### 3.2. Investigations on Mortars

**Figure 5:**
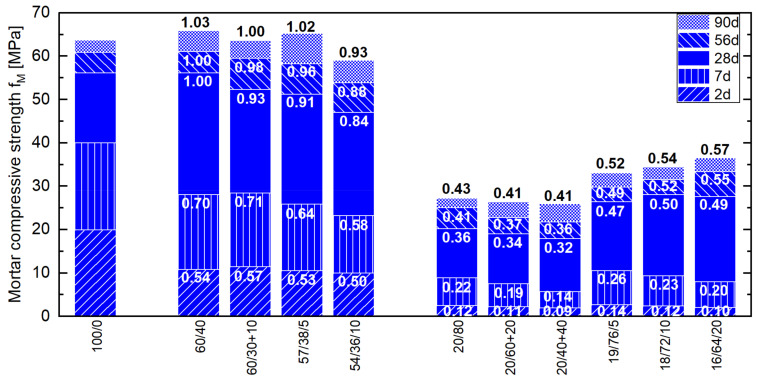
Compressive strengths f_M_ of the mortars with different binder compositions at 2, 7, 28, 56 and 90 days according to DIN EN 196-1 [59]. The values in the columns are the Activity Indexes AI_M_ at the various ages.

The compressive strengths of the different binder compositions measured on mortar at 2, 7, 28, 56 and 90 days are shown in Figure 5 together with their Activity Index AI_M_. The individual values and their relative strength deviations can be found in Supplementary Table S3.

The gain in strength is highest for the reference cement until day 2 and between day 2 and 7, and for the blends between day 7 and 28 At 2 days, the AI_M_ of the blends is below the individual Portland cement proportion. It rises continuously until the 28th day, resulting in a maximum AI_M_ of 1.00 for the “40 wt%” replacements and of 0.50 for the “80 wt%” replacements. Even beyond 28 days, the AI_M_ of the blends can increase by up to 10%. The partial replacement of CT7 by limestone powder causes hardly any changes at an early age, but over time, the strength decreases by several MPa. The addition of hydrated lime permanently reduces the strength of blends with “40 wt%” replacement. In contrast, a small addition (5 wt% hydrated lime) in blends with “80 wt%” replacement has a positive effect on strength at every age, with the improvement being more pronounced after 7 days. Apart from 2 and 7 days, the higher the hydrated lime addition, the greater the impact. Compared to 20/80, the AI_M_ can increase by up to 0.15 (10 MPa) due to the addition of hydrated lime.

Despite the significantly lower 2- and 28-day strengths in some cases, all “40 wt%” blends still meet the strength requirements of class “42.5 N” of the reference cement according to DIN EN 197-1 [85]. The blends with “80 wt%” replacement do not achieve the strengths for “32.5 R” or “32.5N”, but by adding hydrated lime, they fulfill the strength requirements of class “22.5” according to DIN EN 14216 [84]. However, their binder composition is not covered by standards.

### 3.3. Investigations on Concretes

#### 3.3.1. Compressive Strength Tests

**Figure 6:**
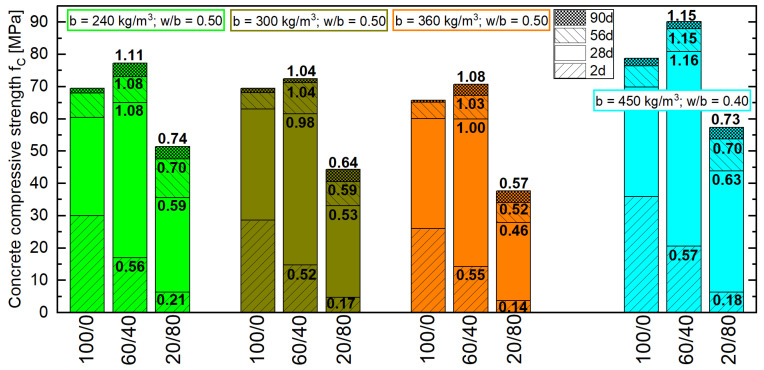
Compressive strengths f_C_ of the concretes with different binder contents and compositions at 2, 28, 56 and 90 days. The values in the columns are the Activity Indexes AI_C_ at the various ages.

**Figure 7:**
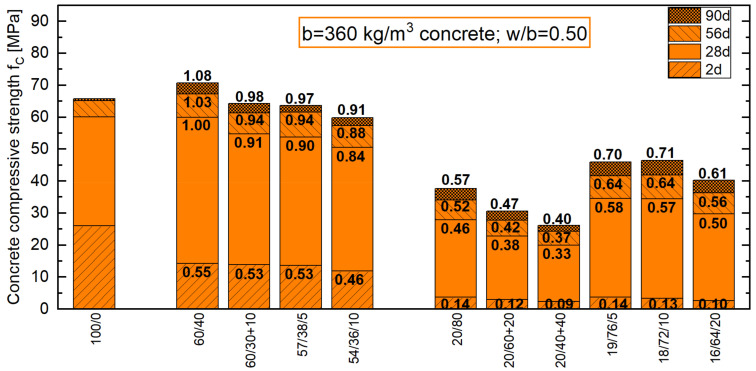
Compressive strengths f_C_ of concretes with different binder compositions (360 kg/m^3^ binder) at 2, 28, 56 and 90 days. The values in the columns are the Activity Indexes AI_C_ at the various ages.

The compressive strengths of the concretes with different binder contents and compositions (Figure 6) and with different binder compositions at 360 kg/m^3^ (Figure 7) are given at 2, 28, 56 and 90 days. Exact values are provided in Supplementary Tables S4 and S6 as well as their relative strength deviations (Supplementary Tables S5 and S6).

Comparing concretes with the same w/b ratio (0.50), the results of Figure 6 are specified as follows. The strengths of the reference concretes are higher than those of 20/80 for every binder content and age. For 60/40, this is only true at 2 days. After that, they exceed the strengths of 100/0 by up to 10%. The Activity Index AI_C_ at 2 days is always below the Portland cement proportion in the binder. By far the greatest strength increase for concretes with calcined clay blends is from 2 to 28 days which is due to the pozzolanic reaction of the CT7. In this way, the low early strength of 60/40 concretes can be compensated until the 28th day, resulting in an AI_C_ of approx. 1.00. After 28 days, the strengths of concretes with the calcined common clay increase more than that of the references (100/0). This is most pronounced at 80 wt%. 60/40 and 20/80 concretes can reach an Activity Index AI_C_ which is 0.50 above the Portland cement proportion by day 90.

At every replacement level and age, the strength increases with decreasing binder content. An explanation is provided in Section 4.2. The effect is particularly pronounced for an 80 wt% replacement by calcined clay.

The concretes with a w/b ratio of 0.40 (b = 450 kg/m^3^) have higher strengths than the other concretes (w/b = 0.50) at every replacement level and age. Reducing the w/b ratio results in a significant rise of the Activity Indexes AI_C_ at both replacement levels.

Figure 7 shows the effects of limestone powder and hydrated lime additions on compressive strengths of concrete made with 360 kg/m^3^ calcined clay blended cement. The replacement of calcined clay by limestone powder yields consistently lower strengths. This trend becomes more pronounced the lower the ratio of CT7 to limestone powder and in particular for later ages, when the strength contribution of the pozzolanic reaction becomes significant.

The addition of hydrated lime is only beneficial for the “80 wt%” replacement. 5 wt% hydrated lime is sufficient to increase significantly the strength from 28 days onwards. At 2 days, the strength remains unchanged. A further 5 wt% addition of hydrated lime has almost no impact. Another increase in the addition (16/64/20) reduces the effect and yields values close to the reference. The pozzolanic reaction of the calcined clay cannot compensate the further reduced amounts of Portland cement and calcined clay. This is also the case with a 5 and 10 wt% addition of hydrated lime at a “40 wt%” replacement of Portland cement.

#### 3.3.2. SEM Images

**Figure 8:**
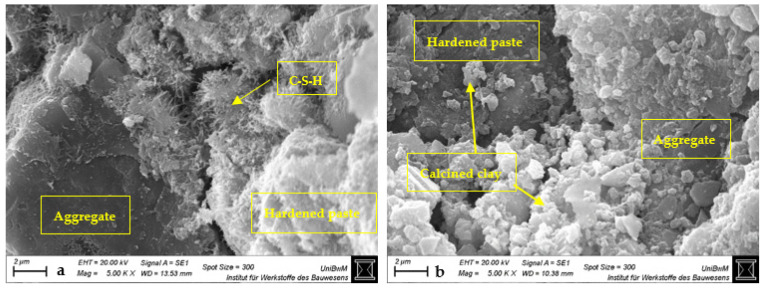
SEM images of concrete (b = 360 kg/m^3^) at 90 days with the binder composition 100/0 (**a**) and 20/80 (**b**) at a magnification of 5000.

SEM images were taken on the interfacial transition zone of concretes (b = 360 kg/m^3^) at 90 days without calcined clay (a) and with 80 wt% calcined clay (b) at a magnification of 5000 (Figure 8). In addition, the magnifications of 2000 and 10,000 at the same spots are in Supplementary Figures S2 and S3.

**Figure A2:**
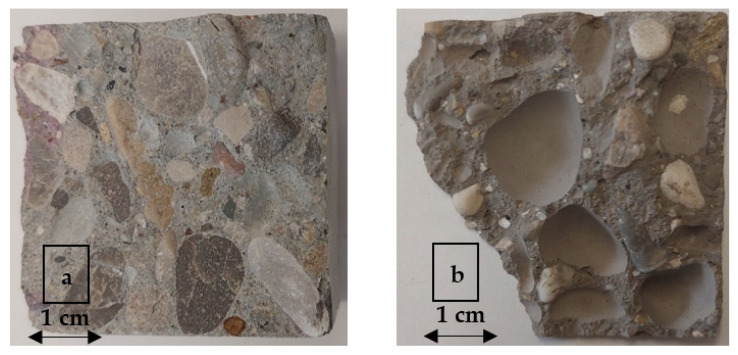
Detailed fragment of concretes (b = 360 kg/m^3^) with the binder composition 100/0 (**a**) and 20/80 (**b**) after tensile splitting test at 90 days.

The interfacial transition zone of the aggregate and hardened paste is clearly visible in both binder compositions. The direct contact between the C-S-H phases and aggregate can be seen in the binder composition 100/0 (Figure 8a). In contrast, hydrate products are more difficult to notice with binder composition 20/80 (Figure 8b). Aggregate and hardened paste are covered with unreacted calcined clay. It appears that a layer of calcined clay covering the aggregate prevents direct contact between the aggregate and hardened paste. This visual perception is confirmed by fragments of the same concretes from the tensile splitting test (Figure A2). With binder composition 100/0 (a) the fracture occurs straight through the aggregate, with binder composition 20/80 (b) at the interfacial transition zone, so that the surface of the aggregate remains intact.

**Figure 9:**
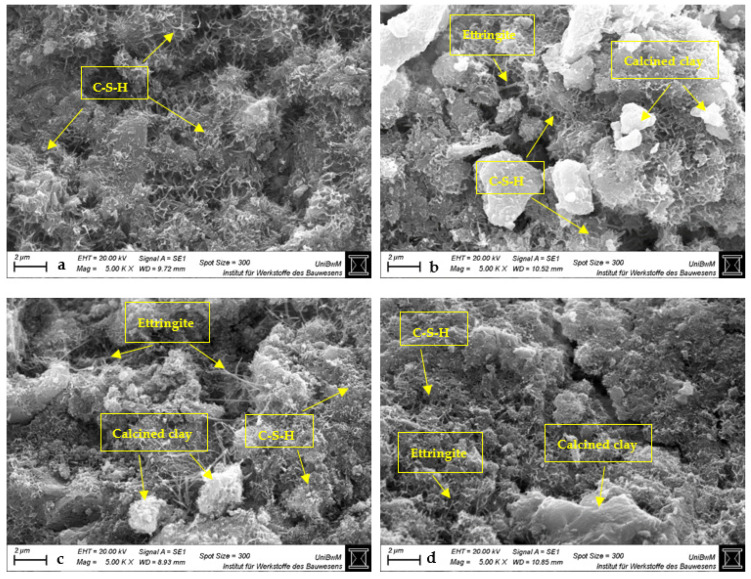
SEM images of concrete (b = 360 kg/m^3^) at 90 days with the binder composition 60/40 (**a**), 20/40 + 40 (**b**), 19/76/5 (**c**) and 16/64/20 (**d**) at a magnification of 5000.

For the concretes 60/40, 20/40 + 40, 19/76/5 and 16/64/20, images of the hardened pastes were also taken. They are shown at a magnification of 5000 in Figure 9 and at a magnification of 10,000 in Supplementary Figure S4. In 60/40 (Figure 9a), no calcined clay particles are visible, as the surface is densely covered by C-S-H phases. In 20/40 + 40, 19/76/5 and 16/64/20 (Figure 9b–d), in addition to calcined clay and C-S-H phases, ettringite is also visible, which is apparently a common hydrate product in the concrete 20/80 (Supplementary Figure S5). In the order of Figure 8b and Figure 9b–d, less unreacted calcined clay is visible for the “80 wt%” replacements.

#### 3.3.3. Carbonation Test

**Figure 10:**
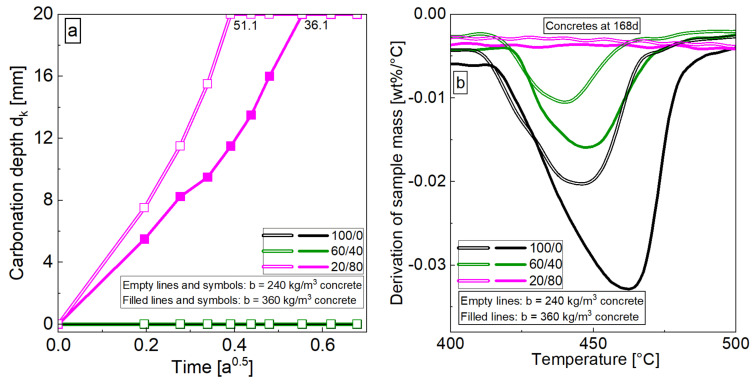
Time-dependent carbonation depth d_k_ under natural conditions until 168 days (**a**) and derivation of sample mass in the thermogravimetric analysis at 168 days (**b**) of concretes with a binder content of 240 (empty lines) and 360 kg/m^3^ (filled lines), each with a binder composition of 100/0, 60/40 and 20/80. The maximum possible carbonation depth is 20 mm. The value at the end of the curve is the carbonation speed k_c_ in mm/a^0.5^. No carbonation was detected for the concretes with a binder composition of 100/0 and 60/40 (overlap of black and green lines).

**Figure A3:**
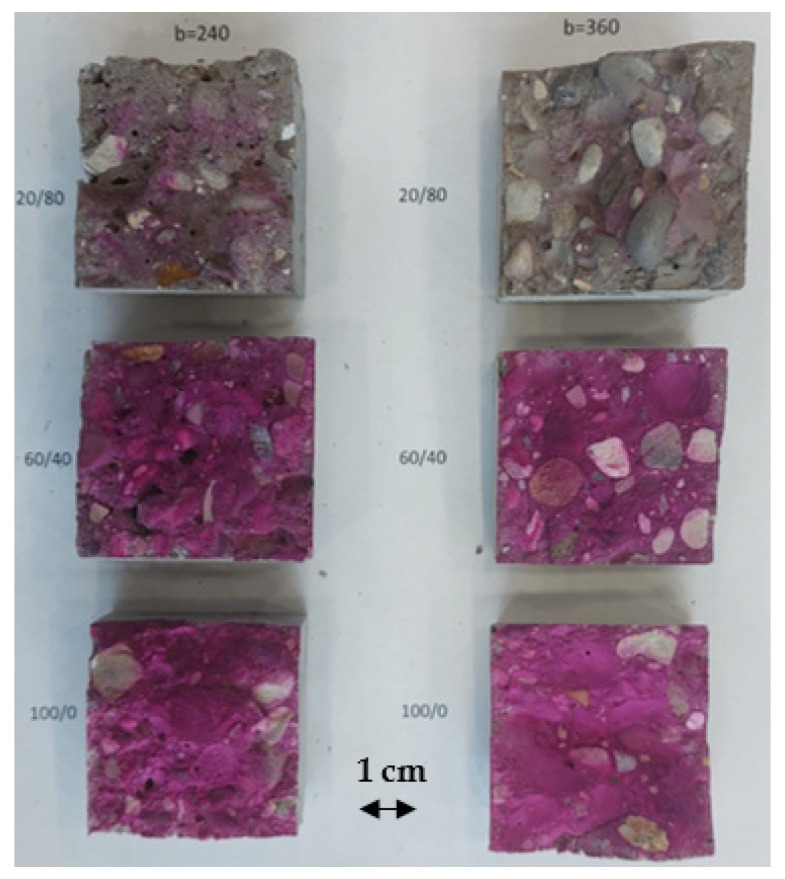
Surfaces of concretes with a binder content of 240 (left row) and 360 kg/m^3^ (right row) sprayed with an indicator solution at 168 days of carbonation. In the bottom row, the binder composition is 100/0, in the middle row 60/40 and in the top row 20/80. Colored areas are alkaline, colorless areas are no longer alkaline due to carbonation.

For binder compositions 100/0, 60/40 and 20/80, the carbonation of concretes with binder contents of 240 and 360 kg/m^3^ is presented as a function of time in Figure 10a (Supplementary Table S7). The surfaces of the various concretes are depicted in Figure A3 sprayed with an indicator solution at 168 days. During the 168-day test period, no carbonation was detected in the 100/0 concretes. The same applies to the concretes with a binder replacement by 40 wt% CT7. But the latter have a lower portlandite content than the reference concretes (Figure 10b) at 168 days. At both binder compositions, this is lower for the smaller binder content because of its lower clinker amount. This is also the reason why concretes with 80 wt% CT7 in the binder carbonate faster at a binder content of 240 than at 360 kg/m^3^. These samples are fully carbonated at 56 and 112 days, which corresponds to a carbonation speed k_c_ of 51 and 36 mm/a^0.5^. The thermogravimetric analysis performed at the end of the carbonation tests (168 days) confirms that the 20/80 concretes contain no portlandite (Figure 10b) as indicated by the lack of mass loss at approx. 450 °C.

#### 3.3.4. Global Warming Potential

**Figure 11:**
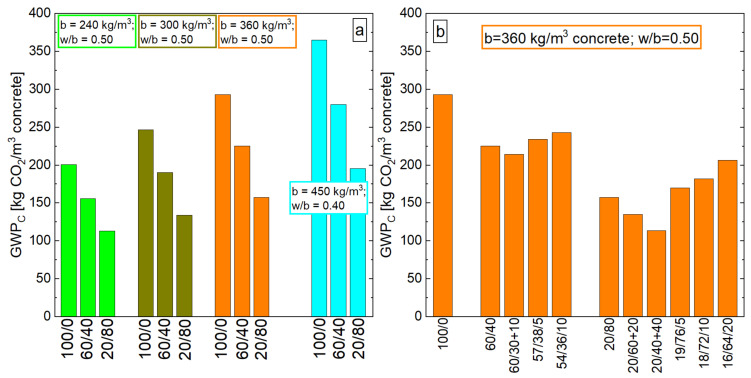
GWP_C_ of the concretes with different binder contents and compositions (**a**) and with different binder compositions at 360 kg binder/m^3^ concrete (**b**).

The calculations of the GWP_C_ of the various concretes were performed using Equation (4) and the results are shown in Figure 11 (Supplementary Table S8). The GWP_C_ decreases with increasing replacement by calcined clay and decreasing binder content, which means that 100/0 concrete with b = 450 kg/m^3^ causes the highest CO_2_ emissions and 20/80 concrete with b = 240 kg/m^3^ causes the lowest (Figure 11a). The GWP_C_ of these two concretes differs by a factor of more than 3. A replacement by 80 wt% calcined clay or a reduction in the binder content from 450 to 240 kg/m^3^ almost halves the GWP_C_. The partial substitution with limestone powder reduces further the GWP_C_ of the different mixes, while hydrated lime has the opposite effect (Figure 11b).

## 4. Discussion

### 4.1. Impact of Calcined Common Clay on Hydration of Blended Cements

Due to its initial filler effect [30] and its adsorption of sulfate carrier [19], the used calcined common clay accelerates the aluminate peak by a few hours, but can still be distinguished from the silicate peak. According to Niemuth [86] and Silva et al. [87] this is sufficient; therefore, no sulfate carrier needs to be added. Panzer et al. [26] found the same for the identical calcined clay using mortar compressive strengths. The high SO_3_ content (Table 2) of the calcined clay makes a sulfate adjustment unnecessary. The aluminate peak occurs at 20 [26], 40 and 80 wt% replacement by calcined clay at the same time (24 h), i.e., it is independent of its replacement level. The increased formation of ettringite (Supplementary Figure S5) consumes portlandite which is responsible for the lower portlandite content of the blends at 2 days (Figure 3), as in [33]. Additionally, ettringite contributes to the early strength due to the porosity reduction [8,15,26].

The pozzolanic reaction of CT7 hardly takes place until 2 days; therefore, almost no portlandite (Figure 3) is consumed and no strength contribution (Figure 5 and Figure 6) is made until then. Despite the increased alkaline environment in the ion solubility test, the Al and Si ion solubilities in CT7 at 20 h are low compared to their final solubilities at 168 h (Table 2). In the cementitious system, Beuntner [15] found higher amounts of reactive ions (Al, Si) for a very similar calcined clay only after 7 days. Consequently, the Activity Index of mortars and concretes at 2 days is notably low and the hydrated lime addition in “80 wt%” replacements is of no benefit at 2 days (Figure 5 and Figure 7). The calcined common clay begins to react significantly pozzolanic after 2 days, since the Activity Indexes of the mortars rise after 2 days and reach higher values than the replacement level for the first time at seven days (Figure 5). The R^3^ test confirms the delayed reactivity of the calcined common clay (Table 2). The pozzolanic reaction of CT7 leads to the decrease in the portlandite content at “40 wt%” replacements over time and to the absence of portlandite at “80 wt%” replacements (Figure 3), because more hydrate phases are formed after 2 days (Figure 2). Thus, the 40 wt% CT7 blend matches the 28-day porosity of the reference cement (Figure 4a), although various calcined clays increase porosity at 2 days [8,33]. It is known that the 28 day-porosity often remains unchanged at low replacement by calcined clay [15,46,47,88] and increases at very high replacement [29].

CT7 consists of approx. 1/3 inert components (Table 2) which reduce the reactive binder content. The replacement of Portland cement by 80 wt% ensures that 27 wt% of the binder is inert. This is the main reason why w/b_eff_ increases as the replacement level of CT7 rises (Table A1 and Table A2). For the “80 wt%” replacements, the high w/b_eff_ contributes to the low hydration heat (Table 6), low amount of bound water (Figure 2), high porosity (Figure 4a) and low compressive strength of mortars (Figure 5) and concretes (Figure 6 and Figure 7). The high porosity and the lack of portlandite (Figure 3 and Figure 10b) result in rapid carbonation of the concretes with 80 wt% CT7 in the binder (Figure 10a). Various studies [42,43,44,45] reported that the carbonation degree of concrete with calcined clay blended cements is higher than that with Portland cement. This effect increases with rising replacement by calcined clay.

The 7 wt% limestone contained in CT7 (Table 2) is sufficient to achieve the synergy of limestone powder and calcined clay on its own [10,19,22], since the amount of AFm phases upon replacements of CT7 by limestone powder does not change (Figure 2). Instead of converting ettringite to monosulfoaluminate, C_3_A or the Al ions from the calcined clay react with the calcium carbonate and portlandite to hemi- and monocarboaluminates after the sulfate carrier is consumed [15,33]. These AFm phases decline porosity and increase the compressive strength, as can be seen from the hydrated lime addition in blends with 80 wt% CT7 (Figure 2, Figure 4a, Figure 5 and Figure 7) and also described in [15,20]. The amount of limestone introduced with CT7 is small compared to that of extra limestone powder used. The replacement by the inert material (Table 2) [89] has a negative effect on the hydration of the binder. Binders with limestone powder show small decreases in hydration heat (Table 6), amount of bound water (Figure 2) and compressive strength of the mortars (Figure 5) and concretes (Figure 7). Due to the absence of a pozzolanic reaction, the portlandite content also increases slightly (Figure 3).

### 4.2. Effect of CT7 Replacement Level on Compressive Strength

**Figure 12:**
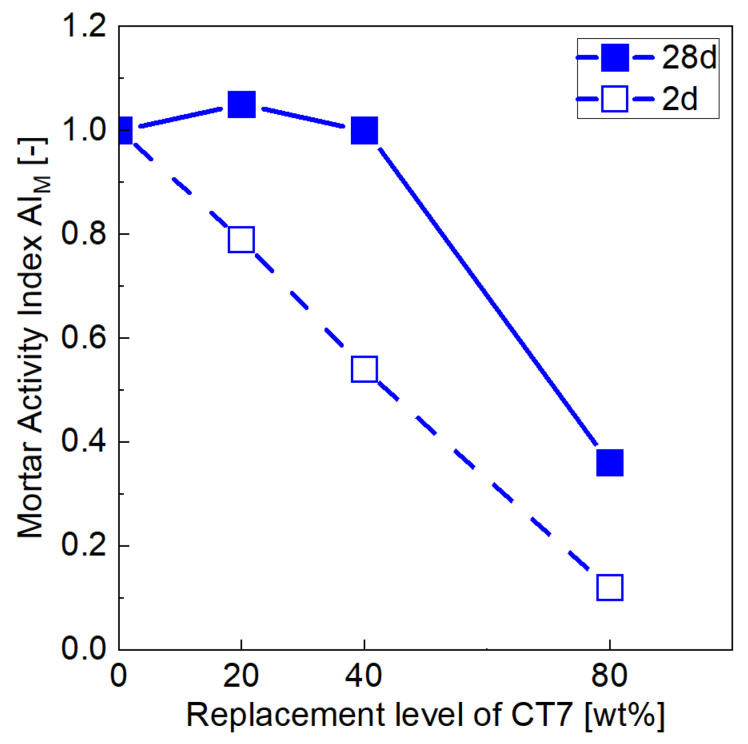
Mortar Activity Index AI_M_ at 2 and 28 days as a function of the CT7 replacement level. The values for a replacement by 20 wt% CT7 are from [26]. Connection lines do not automatically allow for interpolation.

The effects of replacements by the calcined common clay on the mortar compressive strength at 2 and 28 days are shown in Figure 12 using the Activity Index AI_M_. In addition to the values from Figure 5, the values for 20 wt% CT7 replacement from [27] are included. While at 2 days the AI_M_ continuously decreases to below 0.20 with 80 wt% CT7, it reaches at 28 days a maximum with 20 wt% CT7 before falling to below 0.40 with 80 wt%. It is obvious that the pozzolanic reaction of CT7 begins after 2 days. The reactivity of the calcined common clay increases significantly over time, measured in the R^3^ test and in the ion solubility test (Table 2). With 20 wt% CT7, an optimum of the pozzolanic reaction of CT7 and the reaction of the clinker phases of Portland cement is achieved at 28 days. Above 40 wt% CT7, the portlandite provided by the clinker is lacking and limits the pozzolanic reaction, remaining unreacted CT7.

Mortars based on LC^3^ systems are shown to reach 28-day compressive strengths comparable to those of Portland cement references [22,29,35]. In contrast, their 2-day strengths decreased by 40–50%. The same behavior at 2 and 28 days is observed for the mortars in this study containing 40 wt% calcined common clay (Figure 12). A further reduction in clinker proportion to 20 wt%, by substituting calcined kaolinitic clay and limestone powder led to 50–70% lower 28-day strengths (approx. 20 MPa) [29,37], which is in the same range as mortars with 80 wt% calcined common clay in this study (Figure 12). The addition of hydrated lime in a binder with 70 wt% calcined kaolinitic clay increased the 28-day compressive strength of the mortar by approx. 5 MPa [36], which is slightly lower compared to this study (Figure 5).

**Figure 13:**
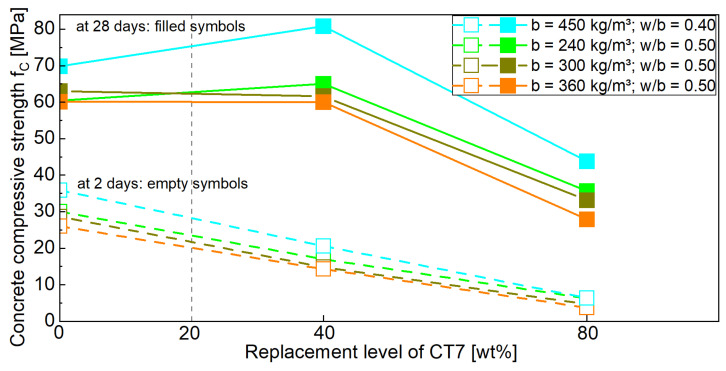
Compressive strength f_C_ [MPa] of various concrete compositions at 2 (empty symbols) and 28 days (filled symbols) as a function of the CT7 replacement level. Connection lines do not automatically allow for interpolation.

Figure 13 shows the concrete compressive strengths at 2 and 28 days (Figure 6) as a function of the replacement levels of CT7. Based on Figure 12, an interpolation between 0 and 40 wt% CT7 is possible at 2 days. But this is not feasible at 28 days, as a maximum compressive strength is reached at 20 wt% (Figure 12). The transfer of strength from mortar to concrete is demonstrated in the following section. Similar to the concretes with 40 wt% calcined common clay in this study, the concretes with similar proportion of calcined kaolinitic clay and limestone powder achieved the compressive strength of the reference concretes (with Portland cement) at 28 days [34,35,90]. At this age, the highest concrete strengths were reached with 15–25 wt% calcined clay in the binder [42,90], which is confirmed in mortar by Figure 12. However, the concrete strength losses at 2 days are approx. as high as the replacement levels [34,42,90] and thus identical to this study.

At a w/b ratio of 0.50, the increase in strength at 2 and 28 days with decreasing binder content is clearly visible. It was also stated in [34,91] that the highest binder content of an LC^3^ does not provide the highest concrete compressive strength. Their optimal binder content was in the range of 240 kg/m^3^. Bonzel [92] provided an explanation for concrete strengths regarding the thickness of the hardened blended cement paste layer in the concrete. With a lower amount of binder, the thickness of the layer decreases which leads to a higher concrete compressive strength.

In addition, the Activity Indexes in Figure 6 show that the impact of calcined common clay increases as the binder content decreases. This finding becomes even more apparent as the replacement level increases.

In this study, reducing the w/b ratio of concretes to 0.40 increases the strength, more so at 28 days than at 2 days. Compared to concretes with 360 kg binder/m^3^, their Activity Indexes are higher (Figure 6) which indicate a more pronounced impact of the calcined common clay in concretes made with a low w/b ratio. This effect intensifies with increasing replacement levels.

### 4.3. Enhancement of Pozzolanic Reaction by Hydrated Lime

The addition of hydrated lime only proves useful in blends with 80 wt% CT7. Various methods show that 20 wt% Portland cement is not sufficient for a complete pozzolanic reaction of 80 wt% CT7. Considering the values at 100/0 and 60/40, the hydration heat (Table 6), amount of bound water (Figure 2) and compressive strength of mortars (Figure 5) and concretes (Figure 6) are lower than expected at 20/80. The opposite is the case for the porosity (Figure 4a). In addition, no portlandite is present for 20/80 after 2 days (Figure 3), and a significant amount of unreacted CT7 in the 20/80 blend is still visible at 90 days (Figure 8b).

The addition of hydrated lime improves the aforementioned properties of the 20/80 blends, although it reduces the amount of Portland cement and calcined clay in the binder. The hydrated lime addition results in significantly more AFm phases after 2 days (Figure 2), which reduce the porosity (Figure 4a) and increase the strength of mortars (Figure 5) and concretes (Figure 7). Dissolved Al ions from the calcined common clay (Table 2), which previously could not react due to the lack of portlandite, utilize the hydrated lime addition together with the limestone from the CT7. This is consistent with the literature [29], as the addition of hydrated lime to metakaolin blends, which have a portlandite deficit due to the high replacement level of 60 to 80 wt%, increases the reaction degree of the metakaolinite, the amount of hemi- and monocarboaluminates and finally the mortar compressive strength. An increasing reaction degree is also observed for CT7 in SEM images. The amount of unreacted CT7 (Figure 8b) decreases with hydrated lime addition (Figure 9c,d).

Sun et al. [29] were also able to increase the compressive strength after a few days by adding hydrated lime to limestone calcined clay cements with less than 50 wt% clinker. The lower their clinker content, the greater is the benefit of adding hydrated lime since it increased the reaction degree of the calcined clay. The addition of hydrated lime to a binary binder (calcined clay cement) with a clinker content below 50 wt% also increases strengths [36,93].

Furthermore, the addition of hydrated lime delays the aluminate peak of calcined clay blended cements, as is the case when added to Portland cement [94]. The rapid release of Ca^2+^ and OH^−^ ions slows down the dissolution-precipitation equilibrium of cement. The hydration heat until 2 days decreases continuously with the addition of hydrated lime in blends with “80 wt%” replacement, but a slight increase is observed thereafter (Table 6). This shows once again that the CT7 only reacts significantly pozzolanic after 2 days. Despite the presence of portlandite, a reaction partner is missing because hardly any Al and Si ions are dissolved from CT7 until 2 days. The increased formation of ettringite caused by the SO_3_ contained in the CT7 is the reason that the 60/40 and 20/80 blends achieve a 2-day hydration heat of 80% and 40% relative to the reference cement, i.e., each 20% higher than their clinker proportion.

In the 20/80 blends, the ratio of gel to microcapillary pores is very small compared to the blends without and with “40 wt%” (Figure 4a) because CT7 is partially unreacted and fewer hydrate phases are formed. The CT7 as solid has a high and coarse porosity (Figure 4b), while hydrate phases reduce porosity and refine the pore structure [15]. The addition of hydrated lime increases the ratio of gel to microcapillary pores (Figure 4a), since less unreacted calcined clay and more hydrated phases are present.

### 4.4. Correlation Between Microstructural Properties and Compressive Strengths

**Figure 14:**
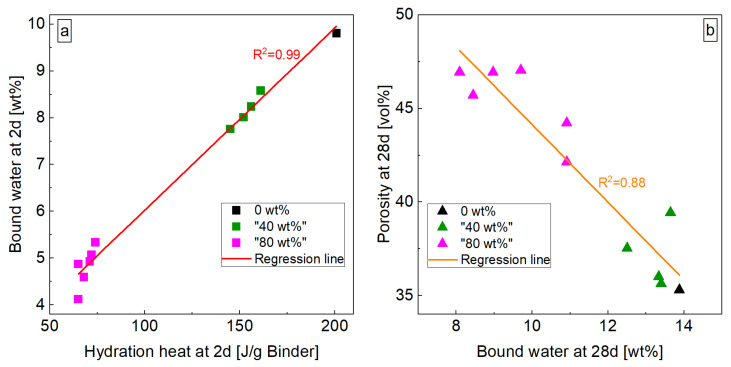
Bound water as a function of hydration heat at 2 days (**a**) and porosity at 28 days (**b**). The different binder compositions are grouped according to their replacement level (Table 3).

The correlation between hydration heat (Table 6) and bound water (Figure 2) at 2 days and between bound water and porosity (Figure 4) at 28 days is presented in Figure 14a,b. The coefficients of determination R^2^ for the various binder compositions show very high correlations [95]. The lower the water binding of pastes, the lower their hydration heat and the higher their porosity. This relationship becomes more pronounced as the replacement level increases.

**Figure 15:**
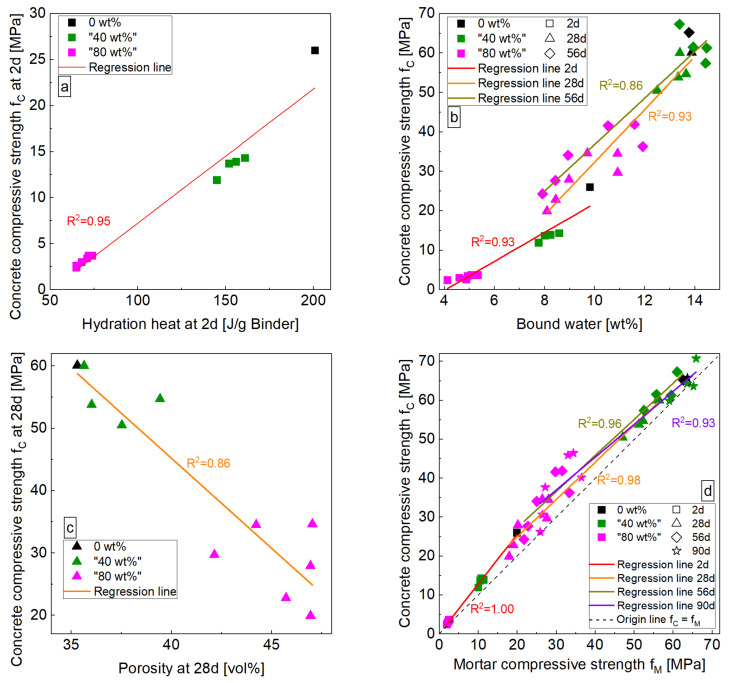
Concrete compressive strength f_C_ (360 kg binder/m^3^) as a function of the hydration heat at 2 days (**a**), bound water content at 2, 28 and 56 days (**b**), porosity at 28 days (**c**) and mortar compressive strength at 2, 28, 56 and 90 days (**d**). The different binder compositions are grouped according to their replacement level (Table 3). Regression lines are formed for every age.

Microstructural investigations yield the underlying information which help to interpret the results of the compressive strength tests of mortars and concretes. The correlation of the early paste hydration heat to the early concrete compressive strength (b = 360 kg/m^3^) in Figure 15a is very high (R^2^ = 0.95) [95]. With increasing replacement level, hydration heat and strength decrease. The missing reaction of the calcined common clay at early age enables an application for the blends as very low heat special cement [84], especially for the blends with “80 wt%” CT7.

Also, the correlation between bound water of paste and concrete compressive strength (b = 360 kg/m^3^) is very high at 2, 28 and 56 days (Figure 15b) [95]. The small amount of hydrate phases explains the low strengths of the concretes and mortars with “80 wt%” CT7 in the binder. The nearby regression lines at 28 and 56 days illustrate that the majority of the reaction mechanisms in the binder are completed at 28 days. Both regression lines exhibit a steeper slope than at 2 days, indicating that the strength gain outpaces the increase in water binding at later ages.

The correlation between paste porosity and concrete compressive strength (b = 360 kg/m^3^) at 28 days (Figure 15c) is high (R^2^ = 0.86) [95]. The low strengths of concretes with “80 wt%” replacements are plausible, since strength decreases with increasing porosity [96], triggered by their low water binding (Figure 14b).

The compressive strengths and Activity Indexes of the mortars without and with “40 wt%” CT7 (Figure 5) are very similar to those of the concretes (Figure 7). But the mortars with an “80 wt%” replacement level have lower strengths and AI_M_ than the concretes from the 28th day onwards. This is well visible in the correlation of mortar and concrete compressive strengths (b = 360 kg/m^3^) in Figure 15d, which is very high for every age [95]. The curve of the measurement points is slightly bent in favor of higher concrete compressive strengths for the “80 wt%” replacements (at 28, 56, 90 days). The reasons for this are as follows.

A lack of portlandite in the hardened paste has an influence on the concrete compressive strengths through the interfacial transition zone [97,98]. Due to the “80 wt%” replacement of Portland cement by the calcined common clay, there is no longer any portlandite that adheres to the aggregates in the interfacial transition zone [99]. This effect results in higher strengths in concretes than in mortars [100,101,102], as described in Section 3.2. It is also conceivable that the bond between the aggregates in mortar is worse than in concrete, because the reduced amount of C-S-H phases in the “80 wt%” replacement is not so sufficient for the surface area of the aggregate, which is many times higher in mortar than in concrete [103,104].

The interfacial transition zone also serves as an explanation for the concrete strength decrease at a later age with higher hydrated lime addition in the “80 wt%” replacement (Figure 7), since the mortars behave in an opposite way (Figure 5). Apart from ettringite formation, the addition of hydrated lime in the “80 wt%” replacements mainly increases the AFm phases (hemi- and monocarboaluminates) and less the C-S-H phases (Figure 2); therefore, the interfacial transition zone in the concrete is deteriorated. In the mortar, the impact of the interfacial transition zone on the strength is not as important and the many AFm phases promote the bond between the fine aggregates (high surface area).

### 4.5. Assessment of Concrete Compressive Strengths in Terms of Sustainability

**Figure 16:**
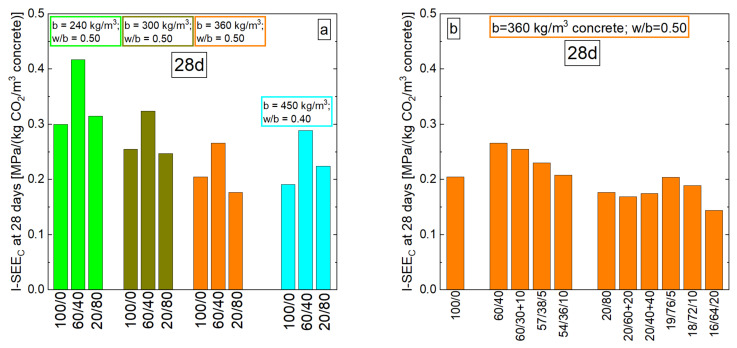
I-SEE_C_ at 28 days of the concretes with different binder contents and compositions (**a**) and with different binder compositions at 360 kg binder/m^3^ concrete (**b**).

The concrete compressive strengths f_C_ at 28 days are sustainably weighed (Figure 16) considering the GWP_C_ (Figure 11) and using Equation (5). The resulting Integrated Strength Eco-Efficiency (I-SEE_C_) is always highest for the 60/40 concretes (Figure 16a). Their strengths are at the level of 100/0 concretes, but have lower GWP_C_. While the I-SEE_C_ of 20/80 is lower than that of 100/0 for concrete with 360 kg binder/m^3^, the opposite is found for 240 kg binder/m^3^. The latter is also the case for the concrete with 450 kg binder/m^3^ (w/b = 0.40). The replacement of calcined clay by limestone powder hardly changes the I-SEE_C_, as limestone powder lowers both, strength values and CO_2_ emissions (Figure 16b). The addition of hydrated lime only pays off in terms of I-SSE_C_ at small percentages in the “80 wt%” replacement (19/76/5) where it provides the partner needed for the pozzolanic reaction. With further addition, the I-SEE_C_ decreases in the same way as with an addition in “40 wt%” replacement, because the GWP_C_ rises as the strength declines.

**Figure A4:**
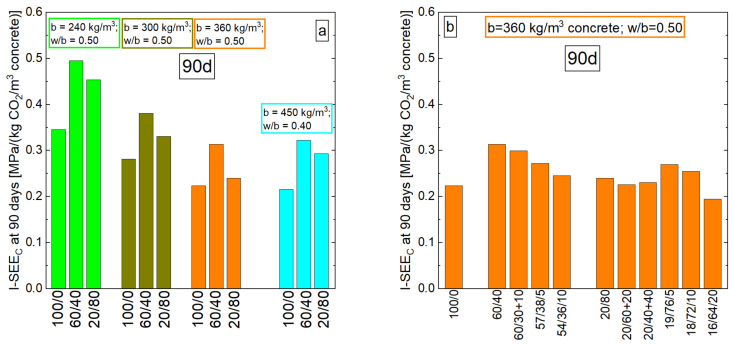
I-SEE_C_ at 90 days of the concretes with different binder contents and compositions (**a**) and with different binder compositions at 360 kg binder/m^3^ concrete (**b**).

At 90 days, all concrete made with calcined clay blends have higher I-SEE_C_ than the reference (Figure A4) due to the still progressing pozzolanic reaction. This is particularly noticeable for concretes with “80 wt%” binder replacement (Figure A4a).

**Figure A5:**
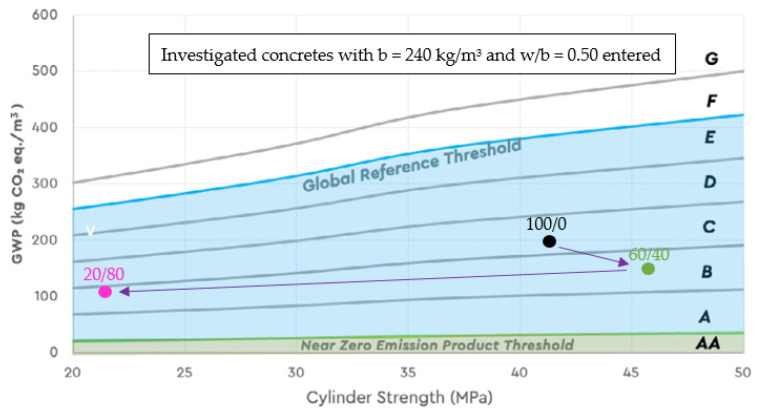
Concrete compressive strengths at 28 days with 0, 40 and 80 wt% calcined clay (240 kg binder/m^3^ concrete) banded by GWP_C_ [40].

The Global Cement and Concrete Association provides a global definition for low carbon concrete [40]. The cylinder compressive strength of concrete is assessed using the GWP_C_ and divided into different bands. In Figure A5, the concretes with 240 kg binder/m^3^ at the various replacement levels are classified as exemplary by their converted cylinder strengths and GWP_C_. Through replacements by 40 and 80 wt% calcined common clay, the concretes reach the band B, although the conventional concrete (100/0) already performs well with band C.

The Concrete Sustainability Council defines different levels of reduction in CO_2_ emission compared to an industry benchmark, which varies depending on the concrete strength class [41]. While the 100/0 concrete with 240 kg binder/m^3^ (C40/50) meets Level 1 (>30% reduction in CO_2_ emission), a 40 wt% replacement by CT7 (C45/55) yields an improvement to Level 3 (>50% reduction) and an 80 wt% replacement (C20/25) to Level 2 (>40% reduction).

## 5. Conclusions

In this study, pastes, mortars and concretes using up to 80 wt% calcined common clay as binder component were examined. The following conclusions can be drawn:

- Since the pozzolanic reaction of the calcined common clay takes place mostly after 2 days, the 2-day Activity Indexes of concretes with CT7 are smaller than their Portland cement proportion. But from day 28 onwards, concretes with 40 wt% calcined clay reach at least the same strength as the references. A further increase up to 80 wt% replacement results in partially unreacted calcined clay and at least one-third lower strength value.
- The correlations from microstructural properties to the concrete compressive strength are very high and establish a causality. From day 28 onwards, the binders without and with 40% calcined clay have the same amount of bound water and porosity. But at the same time, binders with 80 wt% calcined clay exhibit a one-third reduction in hydrate phases, resulting in one-third lower porosity.
- The sulfate naturally present in the clay causes perfect sulfation of the calcined clay blends, which affects their early hydration through the formation of ettringite. Compared to the reference at 2 days, the binders with 40 and 80 wt% calcined clay have only a maximum of 20 and 60% less hydration heat and hydrate phases.
- Carbonation is only observed in concretes with 80 wt% calcined clay which progresses very fast due to the lack of portlandite and the high porosity. The potential for carbonation increases as the binder content decreases, since the portlandite content decreases.
- The GWP of concretes decrease with a 40 or 80 wt% calcined clay replacement by almost 25 or 50%. Thus, the best strength eco-efficiencies at 28 days are achieved with 40 wt% calcined clay in the binder. With 80 wt%, they are on a level with the reference cement.
- The reduced strength of concrete containing limestone powder is offset by its lower GWP. Hydrated lime must be used with caution in binders due to its high GWP. A 5 wt% addition of hydrated lime increases the strength of the concrete with 80 wt% calcined common clay by almost 10 MPa sustainably, as the calcined clay now has sufficient reaction partner. With a replacement by 40 wt% calcined common clay, enough portlandite is ensured by the clinker reaction.

## Figures and Tables

**Table 1:** Mineralogical composition (provided by the producer), chemical composition and physical parameters of the cement used.

Mineralogical Composition [wt%]		Chemical Composition [wt%]		Physical Parameters	
C_3_S	60.4	SiO_2_	20.1	Particle density [g/cm^3^]	3.15
C_2_S	18.2	Al_2_O_3_	5.2	BET SSA [m^2^/g]	1
C_3_A	6.5	Fe_2_O_3_	2.8	d’ [µm]	31.9
C_4_AF	8.3	CaO	62.4	Water demand [%]	27
MgO	0.9	MgO	1.5	Blaine SSA [cm^2^/g]	2742
CaO	0.4	Na_2_O	1.5	f_2d_ [N/mm^2^]	19.9
CaCO_3_	0.8	K_2_O	1.9	f_28d_ [N/mm^2^]	56.1
CaSO_4_	1.5	TiO_2_	0.1		
CaSO_4_·0.5H_2_O	0.7	SO_3_	2.8		
CaSO_4_·2H_2_O	0.2	LOI	1.8		
Minor components	2.1				

**Table 2:** Mineralogical and chemical composition, physical parameters and reactivity of the common clay (CT7), limestone powder (LSP) and hydrated lime (CH).

Mineralogical Composition [wt%]				Chemical Composition [wt%]				Physical Parameters and Reactivity					
	Raw Clay	LSP	CH		CT7	LSP	CH				CT7	LSP	CH
Kaolinite	23			SiO_2_	50.6	4.3	1.3	Particle density [g/cm^3^]			2.60	2.70	2.25
Smectite-Illite	32			Al_2_O_3_	21.8	0.2	0.3	BET SSA [m^2^/g]			6	2	13
Illite	5			Fe_2_O_3_	8.0	0.1	0.2	Particle diameter [µm]		d_10_	1.9	0.7	1.5
Chlorite	6			CaO	5.9	52.8	71.1			d_50_	12.5	4.0	6.9
Calcite	7	96	15	MgO	2.7	0.3	0.5			d_90_	33.8	23.3	28.4
Quartz	20	4		Na_2_O	1.1	0.5	0.7	Water demand [%]			39	19	72
Hydrated lime			80	K_2_O	5.5	0.1	0.2						
Others	7		5	TiO_2_	1.0	0.0	0.0	Cumulative heat release [J/g]		24 h	146	7	
				SO_3_	1.9	0.1	0.1			168 h	427	11	
				LOI	1.6	41.5	25.7	Ion solubility [mmol/L]	20 h	Al	1.34	0.0	
										Si	2.63	0.1	
									168 h	Al	5.33		
										Si	8.32		

**Table 3:** Relative compositions of the binders for blended cement pastes, mortars and concretes.

Replacement Level	Binder Composition	CEM I 42.5 N [wt%]	CT7 [wt%]	LSP [wt%]	CH [wt%]	Total [wt%]
0 wt%	100/0	100	0	0	0	100
“40 wt%”	60/40	60	40	0	0	100
	60/30 + 10	60	30	10	0	100
	57/38/5	57	38	0	5	100
	54/36/10	54	36	0	10	100
“80 wt%”	20/80	20	80	0	0	100
	20/60 + 20	20	60	20	0	100
	20/40 + 40	20	40	40	0	100
	19/76/5	19	76	0	5	100
	18/72/10	18	72	0	10	100
	16/64/20	16	64	0	20	100

**Table 4:** Compositions (water-to-binder ratio, binder content) of the pastes, mortars and concretes.

	w/b	0 wt% Repl.	“40 wt%” Replacement			“80 wt%” Replacement		
Binder Composition		100/0	60/40	60/30 + 10	57/38/5 54/36/10	20/80	20/60 + 20 20/40 + 40	19/76/5 18/72/10 16/64/20
Paste	0.50	<-------------------------- Made for each binder composition -------------------------->						
Mortar	0.50	<-------------------------- Made for each binder composition -------------------------->						
Concrete	0.50	<------------------------------------------- b = 360 kg/m^3^ ------------------------------------>						
		b = 300 kg/m^3^ b = 240 kg/m^3^	b = 300 kg/m^3^ b = 240 kg/m^3^	- - - -		b = 300 kg/m^3^ b = 240 kg/m^3^	- - - -	
	0.40	b = 450 kg/m^3^	b = 450 kg/m^3^	- -		b = 450 kg/m^3^	- -	

**Table 5:** GWP data of materials used without transportation from [13,38,77].

Material	CEM I 42.5 N	CT7	LSP	CH	Aggregate	Water	Superplasticizer
GWP [kg CO_2,e_/kg]	0.7977	0.32	0.026	1.102	0.003	0.0003	0.944

**Table 6:** Hydration heat of the pastes with different binder compositions at 48 h and 168 h.

Replacement Level		0 wt%	“40 wt%”				“80 wt%”					
Binder Composition		100/0	60/40	60/30 + 10	57/38/5	54/36/10	20/80	20/60 + 20	20/40 + 40	19/76/5	18/72/10	16/64/20
Hydration Heat [J/g Binder]	at 48 h	201	161	156	152	145	74	68	64	72	71	65
	at 168 h	306	249	240	236	222	135	120	111	136	134	124
	48–168 h	105	88	84	84	77	61	52	47	64	63	59

## Data Availability

The original contributions presented in this study are included in the article/Supplementary Material. Further inquiries can be directed to the corresponding author.

## Supplementary Materials

The following supporting information can be downloaded at: https://www.mdpi.com/article/10.3390/ma19112322/s1, Figure S1: Particle size distribution of materials used. Figure S2: SEM images of concrete (b = 360 kg/m^3^) at 90 days with the binder composition 100/0 at a magnification of 2000 (a) and 10,000 (b). Figure S3: SEM images of concrete (b = 360 kg/m^3^) at 90 days with the binder composition 20/80 at a magnification of 2000 (a) and 10,000 (b). Figure S4: SEM images of concrete (b = 360 kg/m^3^) at 90 days with the binder composition 60/40 (a), 20/40 + 40 (b), 19/76/5 (c) and 16/64/20 (d) at a magnification of 10,000. Figure S5: SEM image of concrete (b = 360 kg/m^3^) at 90 days with the binder composition 20/80 at a magnification of 20,000. Table S1: Bound water and portlandite content of the hardened pastes with different binder compositions at 2, 28 and 56 days. Table S2: Porosity and its distribution of the hardened pastes with different binder compositions at 28 days and CT7. Table S3: Compressive strength of mortars with different binder compositions at 2, 7, 28, 56 and 90 days. The relative strength deviation indicates the difference between the maximum and minimum strength of a mortar of the same age in relation to its mean value. Table S4: Compressive strength of concretes with different binder contents and compositions at 2, 28, 56 and 90 days. Table S5: Relative strength deviation of concretes with different binder contents and compositions at 2, 28, 56 and 90 days. It indicates the difference between the maximum and minimum strength of a concrete of the same age in relation to its mean value (Supplementary Table S4). Table S6: Compressive strength of concretes (360 kg binder/m^3^) with different binder compositions at 2, 28, 56 and 90 days. The relative strength deviation indicates the difference between the maximum and minimum strength of a concrete of the same age in relation to its mean value. Table S7: Carbonation depth d_k_ [mm] of concretes with a binder content of 360 and 240 kg/m^3^ at different binder compositions. The maximum possible carbonation depth is 20 mm. Table S8: GWP_C_ of concretes with different binder contents and compositions.

## Abbreviations

LC^3^	Limestone calcined clay cement
Al	Aluminum
Si	Silicon
SEM	Scanning electron microscopy
AI	Activity Index
LSP	Limestone powder
CH	Hydrated lime
GWP	Global Warming Potential
I-SEE	Integrated Strength Eco-Efficiency

## Appendix A

**Table A1:** Compositions of the concretes with 360 kg binder/m^3^ (w/b = 0.50). Contents of the materials are given in [kg/m^3^ concrete]. The assumed air content is 1.5 vol%.

	b = 360 kg/m^3^; w/b = 0.50										
Replacement Level	0 wt%	“40 wt%”				“80 wt%”					
Binder composition	100/0	60/40	60/30 + 10	57/38/5	54/36/10	20/80	20/60 + 20	20/40 + 40	19/76/5	18/72/10	16/64/20
Cement	360	216	216	205.2	194.4	72	72	72	68.4	64.8	57.6
Calcined clay	0	144	108	136.8	129.6	288	216	144	273.6	259.2	230.4
Limestone powder	0	0	36	0	0	0	72	144	0	0	0
Hydrated lime	0	0	0	18	36	0	0	0	18	36	72
Water	180	180	180	180	180	180	180	180	180	180	180
Aggregate	1893	1866	1867	1861	1856	1840	1842	1845	1835	1832	1825
Superplasticizer [wt% of binder]	0.00	0.41	0.20	0.35	0.34	0.65	0.38	0.24	0.68	0.67	0.62
w/b [-] ^1^	0.500	0.502	0.501	0.502	0.502	0.504	0.502	0.501	0.504	0.504	0.504
w/b_eff_ [-] ^2^	0.50	0.58	0.63	0.60	0.62	0.69	0.84	1.08	0.71	0.72	0.75

^1^ Water content of superplasticizer is considered. ^2^ Water content of superplasticizer and hydrated lime, and only reactive components (cement, calcined clay minerals, quicklime) of binder are considered.

**Table A2:** Compositions of the concretes with 300 and 240 kg binder/m^3^ (w/b = 0.50) and with 450 kg binder/m^3^ (w/b = 0.40). Contents of the materials are given in [kg/m^3^ concrete]. The assumed air content is 1.5 vol%.

	b = 300 kg/m^3^; w/b = 0.50			b = 240 kg/m^3^; w/b = 0.50			b = 450 kg/m^3^; w/b = 0.40		
Replacement Level	0 wt%	40 wt%	80 wt%	0 wt%	40 wt%	80 wt%	0 wt%	40 wt%	80 wt%
Binder composition	100/0	60/40	20/80	100/0	60/40	20/80	100/0	60/40	20/80
Cement	300	180	60	240	144	48	450	270	90
Calcined clay	0	120	240	0	96	192	0	180	360
Limestone powder	0	0	0	0	0	0	0	0	0
Hydrated lime	0	0	0	0	0	0	0	0	0
Water	150	150	150	120	120	120	180	180	180
Aggregate	2027	2005	1983	2161	2144	2126	1814	1781	1748
Superplasticizer [wt% of binder]	0.51	0.81	1.27	1.36	1.77	3.11	0.27	0.58	1.19
w/b [−] ^1^	0.503	0.505	0.508	0.508	0.511	0.519	0.402	0.403	0.407
w/b_eff_ [−] ^2^	0.50	0.58	0.70	0.51	0.59	0.71	0.40	0.47	0.56

^1^ Water content of superplasticizer is considered. ^2^ Water content of superplasticizer and hydrated lime, and only reactive components (cement, calcined clay minerals, quicklime) of binder are considered.
