# Supplementary material for: Sustainable Cements with up to 80 wt% Calcined Common Clay: Challenges in Microstructure and Compressive Strength of Concretes
Source: Materials (Basel). 2026 May 31;19(11):2322. doi: 10.3390/ma19112322 (PMC13258458; doi:10.3390/ma19112322)
Supplement: Supplementary file 1 [file materials-19-02322-s001.zip › materials-4322294-supplementary.pdf]

materials

MDPI

Supplementary Data

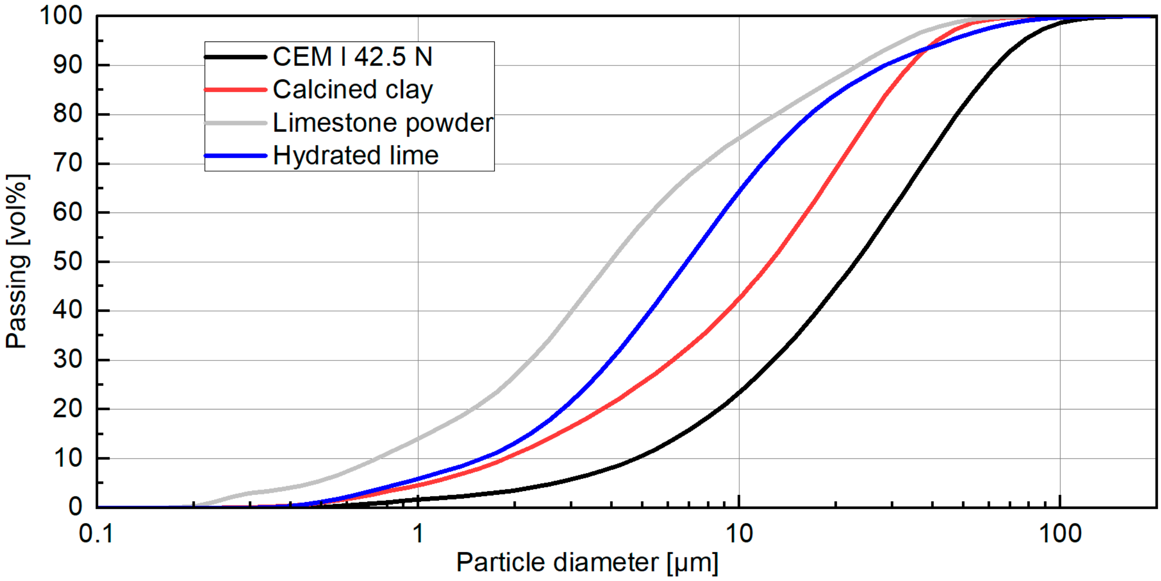

Supplementary Figure S1. Particle size distribution of materials used.

Supplementary Table S1. Bound water and portlandite content of the hardened pastes with different binder compositions at 2, 28 and 56 days.

Binder composition	Age	$H_2O_{25\text{-}140°C}$ [wt%]	$H_2O_{140\text{-}190°C}$ [wt%]	$H_2O_{190\text{-}400°C}$ [wt%]	$H_2O_{25\text{-}400°C}$ [wt%]	$Ca(OH)_2$ [wt%]	$Ca(OH)_2$ [g/100g cement]
100/0	2d	4.76	2.45	2.60	9.81	13.62	13.62
	28d	4.94	3.85	5.09	13.87	17.37	17.37
	56d	3.83	4.11	5.82	13.76	20.71	20.71
60/40	2d	4.34	2.03	2.21	8.58	7.29	12.15
	28d	4.94	3.51	4.96	13.40	6.95	11.58
	56d	3.86	3.84	5.69	13.39	4.66	7.76
60/30+10	2d	4.15	1.94	2.14	8.24	7.49	12.49
	28d	5.02	3.63	5.00	13.65	7.28	12.14
	56d	4.44	4.12	5.91	14.47	4.82	8.04
57/38/5	2d	4.07	1.83	2.11	8.01	9.96	17.48
	28d	4.94	3.45	4.95	13.33	8.29	14.54
	56d	4.20	3.80	5.92	13.93	6.55	11.49
54/36/10	2d	3.86	1.81	2.09	7.76	12.54	23.23
	28d	3.34	3.67	5.49	12.50	10.97	20.32
	56d	4.42	4.02	5.99	14.42	6.47	11.99
20/80	2d	3.19	1.06	1.08	5.34	0.88	4.40
	28d	3.74	2.42	2.81	8.97	0.00	0.00
	56d	3.33	2.54	3.06	8.93	0.00	0.00
20/60+20	2d	2.59	0.96	1.04	4.59	1.37	6.87
	28d	3.43	2.22	2.79	8.45	0.00	0.00
	56d	3.17	2.30	2.97	8.43	0.00	0.00
20/40+40	2d	2.38	0.87	0.87	4.12	1.67	8.35
	28d	3.19	2.16	2.74	8.09	0.00	0.00
	56d	2.77	2.20	2.95	7.92	0.00	0.00

 https://doi.org/10.3390/ma19112322

19/76/5	2d	2.95	1.00	1.13	5.07	3.41	17.94
	28d	3.61	2.64	3.46	9.70	0.00	0.00
	56d	4.01	2.75	3.77	10.53	0.00	0.00
18/72/10	2d	2.77	1.01	1.15	4.93	6.47	35.92
	28d	4.19	2.95	3.77	10.91	0.00	0.00
	56d	4.15	3.15	4.29	11.59	0.00	0.00
16/64/20	2d	2.67	0.96	1.25	4.88	12.16	75.98
	28d	2.89	3.16	4.87	10.92	0.00	0.00
	56d	3.58	3.33	5.01	11.92	0.00	0.00

Supplementary Table S2. Porosity and its distribution of the hardened pastes with different binder compositions at 28 days and CT7

Binder composition	Porosity [vol%]	Air voids [vol%]	Capillary pores [vol%]	Microcapillary pores [vol%]	Gel pores [vol%]	Ratio of gel to microcapillary pores [-]
100/0	35.30	0.31	1.31	2.97	30.71	10.34
60/40	35.63	0.23	1.16	1.97	32.27	16.38
60/30+10	39.42	0.71	2.99	3.07	32.65	10.65
57/38/5	36.01	0.15	1.52	2.22	32.13	14.49
54/36/10	37.52	0.22	0.85	5.75	30.70	5.34
20/80	46.93	0.29	1.43	25.84	19.37	0.75
20/60+20	45.71	0.27	0.95	28.82	15.67	0.54
20/40+40	46.94	0.38	2.14	30.34	14.08	0.46
19/76/5	47.03	0.52	4.10	23.08	19.33	0.84
18/72/10	44.22	0.56	3.20	19.53	20.92	1.07
16/64/20	42.14	0.33	1.50	14.11	26.20	1.86
CT7	53.68	3.08	26.45	19.87	4.29	0.22

Supplementary Table S3. Compressive strength of mortars with different binder compositions at 2, 7, 28, 56 and 90 days. The relative strength deviation indicates the difference between the maximum and minimum strength of a mortar of the same age in relation to its mean value.

Binder	Mortar compressive strength f_M [MPa]					Relative strength deviation [-]				
composition	2d	7d	28d	56d	90d	2d	7d	28d	56d	90d
100/0	19.9	40.0	56.1	62.6	63.7	0.00	0.02	0.05	0.01	0.03
60/40	10.8	28.0	56.1	61.0	65.9	0.05	0.03	0.06	0.03	0.04
60/30+10	11.4	28.4	52.3	59.5	63.6	0.01	0.01	0.05	0.03	0.02
57/38/5	10.5	25.8	51.2	58.2	65.2	0.04	0.01	0.06	0.01	0.02
54/36/10	10.0	23.2	47.0	53.8	59.0	0.01	0.02	0.02	0.05	0.04
20/80	2.5	8.9	20.2	25.0	27.2	0.04	0.02	0.04	0.00	0.01
20/60+20	2.2	7.5	19.0	22.7	26.4	0.05	0.00	0.04	0.02	0.02
20/40+40	1.9	5.7	17.9	21.7	25.9	0.05	0.02	0.05	0.00	0.01
19/76/5	2.7	10.5	26.4	29.8	33.0	0.00	0.02	0.03	0.04	0.05
18/72/10	2.4	9.3	28.0	31.5	34.4	0.06	0.04	0.05	0.02	0.04
16/64/20	2.0	8.0	27.6	33.3	36.5	0.05	0.01	0.05	0.02	0.03

Supplementary Table S4. Compressive strength of concretes with different binder contents and compositions at 2, 28, 56 and 90 days

Binder	300 kg/m³: f_C [MPa]				240 kg/m³: f_C [MPa]				450 kg/m³: f_C [MPa]			
composition	2d	28d	56d	90d	2d	28d	56d	90d	2d	28d	56d	90d
100/0	28.6	63.0	68.1	69.5	30.0	60.4	68.0	69.5	35.9	69.8	76.4	78.7
60/40	14.8	61.6	71.2	72.5	17.0	65.0	73.1	77.3	20.6	80.8	87.9	90.2
20/80	4.7	33.1	40.5	44.4	6.3	35.6	47.6	51.4	6.4	43.8	53.7	57.4

https://doi.org/10.3390/ma19112322

Supplementary Table S5. Relative strength deviation of concretes with different binder contents and compositions at 2, 28, 56 and 90 days. It indicates the difference between the maximum and minimum strength of a concrete of the same age in relation to its mean value (Supplementary Table S4).

Binder composition	300 kg/m³: fc [MPa]				240 kg/m³: fc [MPa]				450 kg/m³: fc [MPa]			
	2d	28d	56d	90d	2d	28d	56d	90d	2d	28d	56d	90d
100/0	0.01	0.02	0.01	0.00	0.06	0.05	0.02	0.01	0.02	0.02	0.04	0.03
60/40	0.06	0.03	0.01	0.01	0.02	0.03	0.02	0.03	0.02	0.03	0.03	0.02
20/80	0.06	0.02	0.01	0.01	0.05	0.05	0.04	0.02	0.08	0.05	0.04	0.01

Supplementary Table S6. Compressive strength of concretes (360 kg binder/m³) with different binder compositions at 2, 28, 56 and 90 days. The relative strength deviation indicates the difference between the maximum and minimum strength of a concrete of the same age in relation to its mean value.

Binder composition	Compressive strength fc [MPa]				Relative strength derivation [-]			
	2d	28d	56d	90d	2d	28d	56d	90d
100/0	26.0	60.1	65.2	65.7	0.06	0.03	0.02	0.01
60/40	14.3	60.0	67.3	70.7	0.06	0.03	0.00	0.01
60/30+10	13.9	54.7	61.3	64.3	0.01	0.03	0.03	0.02
57/38/5	13.7	53.8	61.5	63.6	0.01	0.05	0.00	0.01
54/36/10	11.9	50.5	57.4	59.8	0.03	0.01	0.01	0.00
20/80	3.7	27.9	34.1	37.7	0.05	0.04	0.01	0.00
20/60+20	3.0	22.8	27.7	30.6	0.07	0.02	0.01	0.02
20/40+40	2.4	19.9	24.3	26.2	0.08	0.02	0.01	0.01
19/76/5	3.7	34.6	41.6	45.9	0.05	0.01	0.03	0.02
18/72/10	3.4	34.5	41.9	46.4	0.06	0.02	0.01	0.02
16/64/20	2.6	29.7	36.3	40.2	0.08	0.01	0.01	0.00

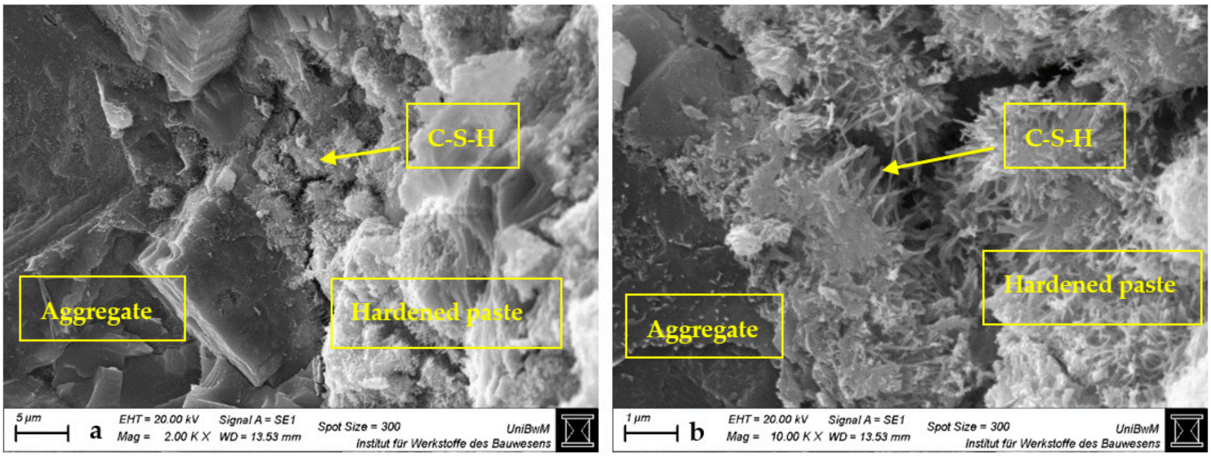

Supplementary Figure S2. SEM images of concrete (b = 360 kg/m³) at 90 days with the binder composition 100/0 at a magnification of 2,000 (a) and 10,000 (b)

https://doi.org/10.3390/ma19112322

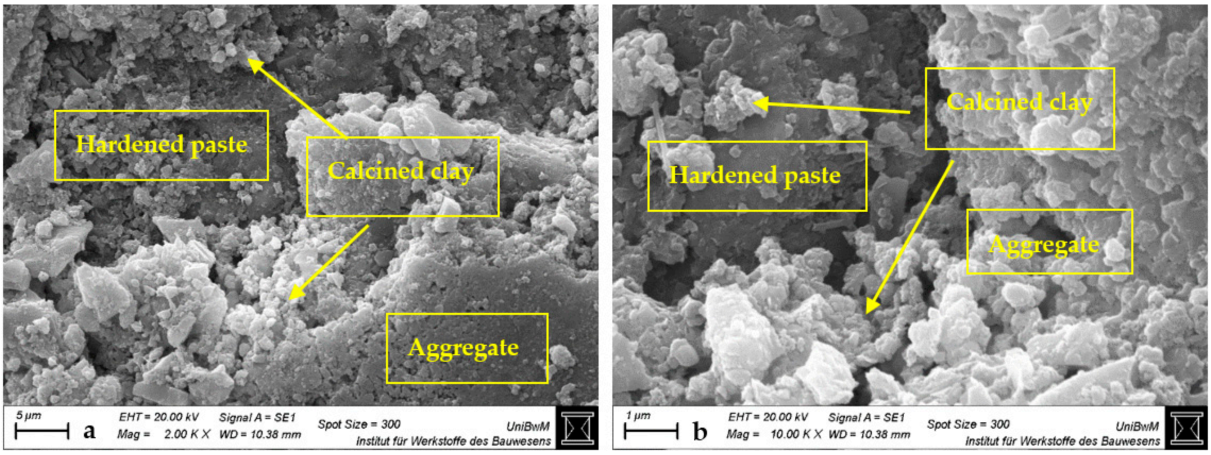

Supplementary Figure S3. SEM images of concrete (b = 360 kg/m^3) at 90 days with the binder composition 20/80 at a magnification of 2,000 (a) and 10,000 (b).

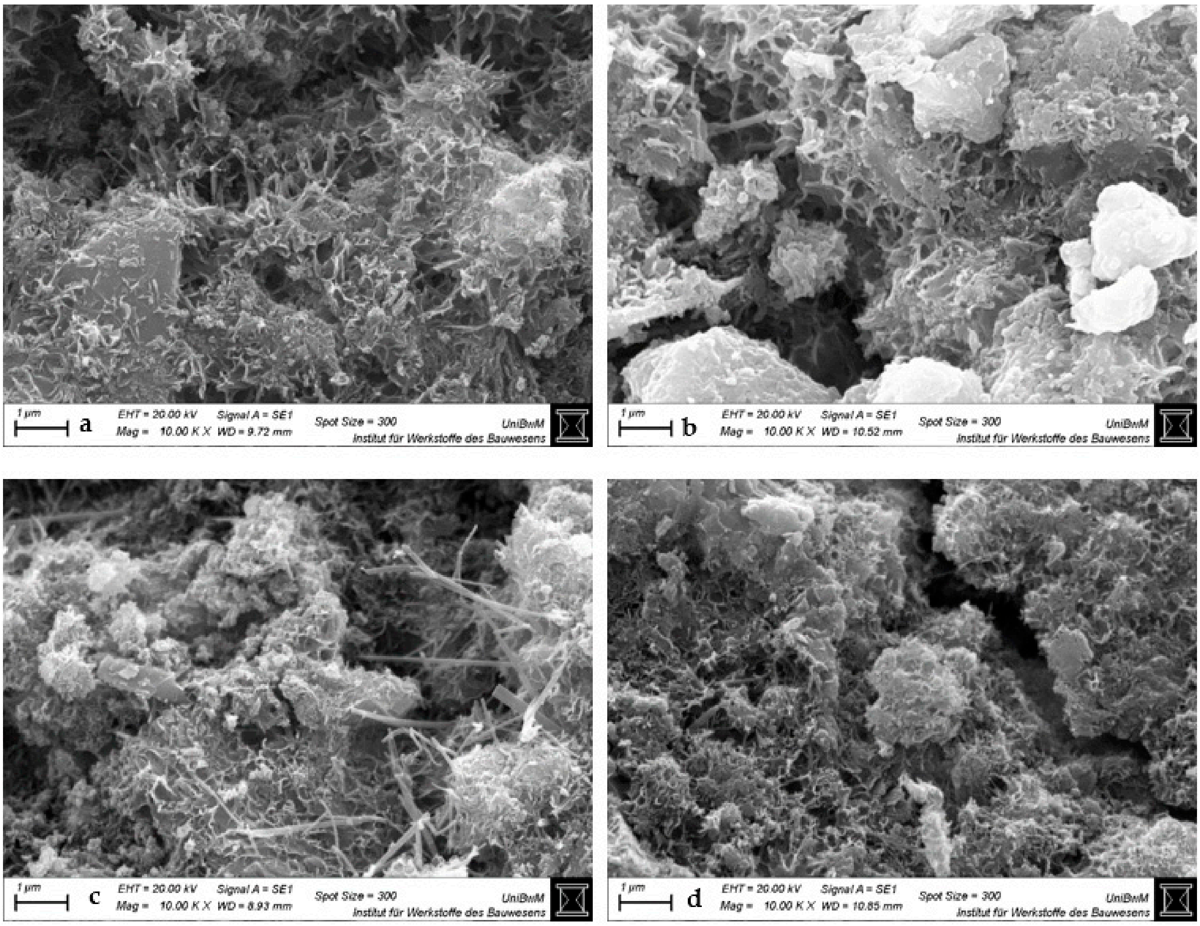

Supplementary Figure S4. SEM images of concrete (b = 360 kg/m^3) at 90 days with the binder composition 60/40 (a), 20/40+40 (b), 19/76/5 (c) and 16/64/20 (d) at a magnification of 10,000.

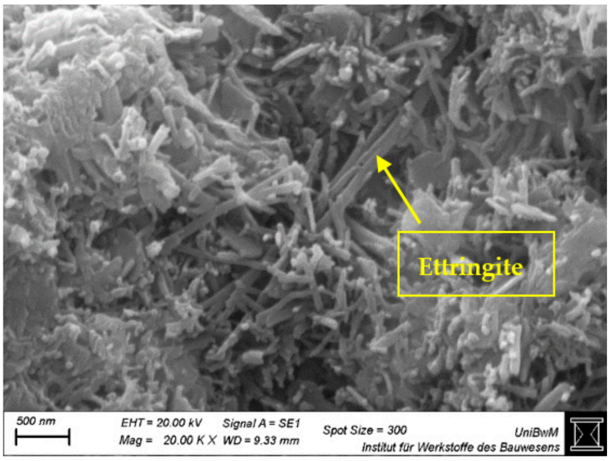

Supplementary Figure S5. SEM image of concrete (b = 360 kg/m³) at 90 days with the binder composition 20/80 at a magnification of 20,000.

Supplementary Table S7. Carbonation depth d_k [mm] of concretes with a binder content of 360 and 240 kg/m³ at different binder compositions. The maximum possible carbonation depth is 20 mm.

Binder composition	b = 360 kg/m³; w/b = 0.50			b = 240 kg/m³; w/b = 0.50		
	100/0	60/40	20/80	100/0	60/40	20/80
t = 0 days	0	0	0	0	0	0
t = 14 days	0	0	5.5	0	0	7.5
t = 28 days	0	0	8	0	0	11.5
t = 42 days	0	0	9.5	0	0	15.5
t = 56 days	0	0	11.5	0	0	20
t = 70 days	0	0	13.5	0	0	20
t = 84 days	0	0	16	0	0	20
t = 112 days	0	0	20	0	0	20
t = 140 days	0	0	20	0	0	20
t = 168 days	0	0	20	0	0	20

Supplementary Table S8. GWP_C of concretes with different binder contents and compositions

Binder composition	GWP_C [kg CO_2/m³ concrete]			
	360 kg binder/m³	300 kg binder/m³	240 kg binder/m³	450 kg binder/m³
100/0	292.9	246.9	201.0	365.2
60/40	225.4	190.3	156.0	280.0
60/30+10	214.1			
57/38/5	234.1			
54/36/10	243.0			
20/80	157.4	134.2	113.2	195.6
20/60+20	135.3			
20/40+40	113.7			
19/76/5	169.8			
18/72/10	182.1			
16/64/20	206.6			

https://doi.org/10.3390/ma19112322
